# Incremental Prognostic Information Beyond Nodal Positivity: The AJCC 8th Edition N1/N2 Subdivision and the Lymph Node Ratio in Resected Periampullary Adenocarcinoma

**DOI:** 10.3390/cancers18203321

**Published:** 2026-10-15

**Authors:** Gun Hyung Na, Changho Seo

**Affiliations:** Department of Surgery, Bucheon St. Mary’s Hospital, College of Medicine, The Catholic University of Korea, 327 Sosa-ro, Wonmi-gu, Bucheon 14647, Republic of Korea; nagh0214@catholic.ac.kr

**Keywords:** lymph node ratio, AJCC 8th edition, nodal staging, nodal yield, periampullary carcinoma, pancreatoduodenectomy, pancreatic cancer, distal cholangiocarcinoma, ampullary carcinoma

## Abstract

**Background/Objectives:** The American Joint Committee on Cancer (AJCC) 8th edition subdivides node-positive periampullary adenocarcinoma into N1 and N2 by the number of positive nodes. We quantified how much this subdivision and the lymph node ratio (LNR) each add to nodal positivity, and how the nodal yield affects the comparison. **Methods:** This retrospective cohort comprised 147 consecutive pancreatoduodenectomies for pancreatic head (n = 49), distal bile duct (n = 63), ampullary (n = 31) or duodenal (n = 4) adenocarcinoma. In covariate-adjusted Cox models stratified by origin, the N1/N2 subdivision and the LNR were tested over nodal positivity and over each other with nested likelihood-ratio tests, overall, with the examined-node count in the model and by the period of resection. **Results:** The median nodal yield was 17 (10 in 2013–2016, 19 in 2017–2024). Beyond nodal positivity, the N1/N2 subdivision added χ^2^ 0.25 (*p* = 0.61; adjusted N2-versus-N1 hazard ratio 1.21, 95% confidence interval [CI] 0.59–2.45) and the LNR added χ^2^ 9.60 (*p* = 0.002; hazard ratio 1.41 per 0.1, 95% CI 1.22–1.62). The LNR added to the N category (*p* = 0.002; optimism-corrected C-index gain 0.020, 95% CI 0.002–0.041), whereas the N category did not add detectably to the LNR (*p* = 0.54). With the examined-node count in the model, the LNR increment was attenuated (*p* = 0.045) and the subdivision’s increment rose (χ^2^ 3.65, *p* = 0.056). From 2017 onward (n = 113), the LNR and the positive-node count were nearly collinear among the node-positive patients (ρ 0.90 versus −0.02 before 2017), and neither the subdivision (*p* = 0.49) nor the LNR (*p* = 0.17) added significantly to positivity. **Conclusions:** The N1/N2 subdivision added little beyond nodal positivity, although imprecisely estimated, and its increment rose when the examined-node count was in the model. The LNR added prognostic information mainly when the yield was low or variable; where the yield was higher and more uniform, the ratio and the count-based category conveyed nearly the same information, and no additional value of the ratio was demonstrated.

## Simple Summary

After surgery for periampullary cancer, the number of lymph nodes containing cancer helps determine the stage. The current system separates node-positive patients into N1 and N2 by the number of positive nodes, but this count depends on how many nodes were examined. In 147 patients at one Korean center, we asked how much the N1/N2 split adds to knowing that any node is positive, whether the lymph node ratio (positive divided by examined nodes) adds information beyond the N category, and how the examined-node count affects these comparisons. The N1/N2 split added little. The ratio added information in the cohort as a whole, but this came mainly from patients resected before 2017, when few nodes were examined. From 2017 onward, when the nodal yield nearly doubled, the ratio and the positive-node count carried almost the same information. The ratio may add most information when nodal yield is low or variable.

## 1. Introduction

Regional lymph node involvement is an adverse prognostic factor after curative-intent resection of periampullary adenocarcinoma, defined here as adenocarcinoma of the pancreatic head, the distal bile duct, the ampulla of Vater or the duodenum [1]. The 7th edition of the American Joint Committee on Cancer (AJCC) staging system recorded only whether any regional node was involved. The 8th edition subdivided node-positive disease into N1 (one to three positive nodes) and N2 (four or more). It applied this count-based category uniformly to the pancreas, the distal bile duct and the ampulla, on the grounds that a number-based categorization “results in better prognostic stratification” [2,3]. The evidence cited comprised two series with medians of 24 and 19 examined nodes [4,5]. The multi-institutional validation that accompanied the change did not evaluate the ratio [6], nor did most later validations [7,8,9,10]. In the Emory series that proposed a revised N classification, the 1–3 versus ≥4 split fitted less well than a 1–2 versus ≥3 split or no split at all [11].

The lymph node ratio (LNR), the number of positive nodes divided by the number examined, accounts for the extent of lymphadenectomy [12,13]. It predicts survival in pancreatic [14,15], distal bile duct [16,17] and ampullary cancer [18], as well as in each of the three main tumor types of a mixed periampullary cohort [1]. In several series, it stratified the prognosis better than the 8th edition category [16,17,19], as did the ratio or the log odds of positive nodes in registry-based comparisons [20,21,22]. To our knowledge, none of these studies tested with nested models whether each descriptor adds information to the other. Three objections have kept the ratio out of staging. First, it depends on the number of nodes examined [4,23]. Second, no threshold has been agreed upon.. The published values range from 0.10 [16] to 0.45 [24], and 0.2 is the most widely used [13,14]. Many were derived within the reporting study by a data-driven search for the best-fitting value [12,15,17,25]. Since in-sample optimization inflates the apparent effect at the chosen value [26], no single threshold transfers readily to another cohort, and the authors of the largest such search treated their value as an indicator rather than a fixed threshold [15]. The optimal value is also said to shift with the nodal yield [5,27]. Third, once the lymph node evaluation is adequate, the count of positive nodes is held to “reflect the true burden of nodal disease” and to be the better indicator [4,28].

These observations leave three questions: how much prognostic information the 8th edition gained by subdividing node-positive disease, whether the ratio adds information that the subdivision does not, and how the nodal yield affects both comparisons. Regarding the first question, validation studies have tested whether N1 and N2 separate upon Kaplan–Meier analysis [7,8,9,10,19], and some have reported the change in discrimination between the two editions [8,10]. None has expressed the subdivision’s contribution as an increment over nodal positivity. Regarding the second question, studies that examined both descriptors did not compare them directly, in part because the two are collinear. Some kept them in separate models [24,29,30] or examined the ratio only within node-positive patients [25]. Others excluded one because of collinearity [1,15,31], or they entered both and reported only the descriptor retained after variable selection [5,17,28,32]. One group cautioned that a model containing both the nodal status and the ratio “cannot give valid results” [31]. Nested likelihood-ratio tests address this concern directly: they compare models with and without a term, and collinearity widens the confidence intervals of the coefficients without invalidating that comparison. We addressed these questions in a single-center cohort in which the median nodal yield nearly doubled during the study period, which allowed the third question to be examined within one institution.

## 2. Materials and Methods

### 2.1. Patients

This retrospective cohort study included consecutive patients who underwent pancreatoduodenectomy with curative intent at our institution between January 2013 and August 2024, identified from the departmental hepato-pancreato-biliary database (Figure S1). Eligible tumors were invasive adenocarcinomas of the pancreatic head, the distal common bile duct, the ampulla of Vater or the duodenum, classified from the synoptic pathology report by site of origin and histological subtype. Excluded were neuroendocrine and mixed adenoneuroendocrine carcinomas, gastrointestinal stromal tumors, adenoma with high-grade dysplasia without invasion, non-ductal pancreatic pathology, hilar cholangiocarcinoma and tumors of another primary site, and patients with no examined lymph node, R2 resection or distant metastasis. Adenosquamous carcinoma (n = 4), a subtype of ductal adenocarcinoma in the World Health Organization classification, was retained and graded as poorly differentiated. Duodenal adenocarcinoma (n = 4) was retained to preserve the periampullary definition [1,33]. Five patients received neoadjuvant therapy and were retained. The one patient who died within 90 days of operation was retained in the primary cohort rather than excluded as perioperative mortality (as had been done in an earlier analysis), because the primary endpoint was death from any cause and exclusion would have conditioned on survival beyond 90 days; exclusion of this patient is reported as a sensitivity analysis (Table S1). Distal and total pancreatectomy were not included because their lymphadenectomy differs from that of pancreatoduodenectomy.

All operations used a standard regional lymphadenectomy, irrespective of tumor origin; no formal change in the institutional lymphadenectomy protocol was documented during the study period. Two surgeons performed 142 of 147 resections. A second hepato-pancreato-biliary surgeon joined the department in late 2016 and initially operated jointly with the first, and a pathologist with a hepato-pancreato-biliary focus joined the department in 2018. The median number of examined nodes rose from 10 in 2013–2016 to 19 in 2017–2024 (from 2017 onward, 24 for the first surgeon and 18 for the second), and specimens with fewer than 10 nodes became uncommon from 2018 onward (15 of 34, 3 of 11 and 5 of 102 specimens in 2013–2016, 2017 and 2018–2024). The relative contributions of surgical dissection and pathological retrieval to this change cannot be separated retrospectively. This study is reported according to the Strengthening the Reporting of Observational Studies in Epidemiology (STROBE) statement [34] and the Reporting Recommendations for Tumor Marker Prognostic Studies (REMARK) [35]. It was approved by the institutional review board as a retrospective review of existing medical records covering the whole study period, and it received a waiver of informed consent.

### 2.2. Nodal Descriptors and Outcomes

The numbers of examined and positive nodes were taken from the synoptic report; nodal stations were not recorded separately. Five descriptors were compared: nodal positivity (any positive node versus none, the 7th edition category); the 8th edition N category, applied by the organ-specific definitions (N1, 1–3 and N2, ≥4 positive nodes for the pancreatic head, the distal bile duct and the ampulla; N1, 1–2 and N2, ≥3 for the duodenum); the number of positive nodes, entered as log(count + 1) because spline analysis suggested nonlinearity; the LNR (reported per 0.1), entered as a linear term after a restricted cubic spline had shown no departure from log-linearity; and the log odds of positive nodes (LODDS), ln[(positive + 0.5)/(examined − positive + 0.5)] [36]. The 8th edition category is nodal positivity plus the N1/N2 subdivision, and the subdivision is treated throughout as the increment of the category over positivity.

Overall survival was measured from resection to death from any cause. Vital status was ascertained from clinical records and, using a survey from September to November 2025, from national health insurance records; survivors were censored at the last date known alive, no later than the administrative closing date of 31 October 2025. Recurrence-free survival was measured to the first of recurrence or death; event-free survivors were censored at the last clinical assessment at which they were known to be recurrence-free, because recurrence is ascertained only at clinic visits. R1 resection was defined as tumor at any inked resection margin (0 mm). Preoperative carbohydrate antigen 19-9 (CA19-9) was the value obtained at diagnostic work-up, a median of 13 days (interquartile range 9–23) before operation. Seventy-six percent of patients underwent preoperative biliary drainage, and the serum bilirubin at the time of measurement was not systematically recorded. CA19-9 was categorized at 200 U/mL, the upper stratum used in pancreatic cancer series [37], because at the conventional 37 U/mL, 71% of measured values were elevated in this largely jaundiced cohort. A separate category was used for the 10 patients in whom it was not measured. Median follow-up was estimated by the reverse Kaplan–Meier method.

### 2.3. Statistical Analysis

Baseline characteristics were compared across tumor origins with the Kruskal–Wallis test and Fisher’s exact test. The reference model was a Cox model with the baseline hazard stratified by tumor origin. It contained age, sex, pT category (T3–4 versus T1–2, as recorded in the synoptic report under the AJCC edition in use at the time; ypT after neoadjuvant therapy), differentiation (poor or undifferentiated versus well or moderate), R1 status, perineural invasion and CA19-9. Covariates were prespecified rather than selected by univariable significance; lymphovascular invasion, which is strongly associated with nodal involvement, was reserved for sensitivity analysis. Postoperative treatment (chemotherapy with or without radiotherapy in 103 patients and radiotherapy alone in three) was recorded as administered or not; since it is decided after pathological staging, it was not entered in the primary models but was examined in sensitivity analyses. Overall stage group was not used because it is a deterministic function of pT and the N category under study. Stratification by origin allows a separate baseline hazard for each origin but assumes a common effect of each covariate. pT, whose definition differs between origins, was therefore treated as an adjustment variable only, and models without pT and with origin-specific pT terms were examined. No a priori sample-size calculation was performed; the cohort comprised all eligible consecutive patients. The reference model had eight coefficients for 77 deaths, and the models containing the N category, LNR or both had 10, 9 and 11.

Each descriptor was added to the reference model, and its contribution was measured by the likelihood-ratio χ^2^ and the change in Akaike information criterion (ΔAIC), a descriptive comparison that penalizes each additional parameter by two units; since the descriptors differ in their number of parameters and are not nested in one another, ΔAIC indicates relative fit and is not a test. The primary analyses were nested likelihood-ratio tests for overall survival with nodal positivity as the common base. They tested the increment of the N1/N2 subdivision over positivity, the increment of LNR over positivity, and, in a model containing both, the increment of each over the other. The last comparison is equivalent to testing the LNR over the N category and the N1/N2 subdivision over positivity plus LNR. The conventional bidirectional comparison of the three-level N category with the LNR is also reported. Since the LNR and the N category are collinear, models containing both were used only for likelihood-ratio tests, and no hazard ratio from such a model is reported. Recurrence-free survival was a secondary endpoint. Departure from log-linearity was tested for LNR and the positive-node count with restricted cubic splines (three knots at the 10th, 50th and 90th percentiles of the node-positive distribution), and the nested tests were repeated with the spline terms. Discrimination was estimated as Harrell’s C-index with bootstrap optimism correction [38]: each model was refitted on 1000 resamples, the mean excess of its C-index on the resample over that on the original cohort was subtracted from the apparent C-index, and differences between models were assessed from the paired per-resample values. The corrected difference is the apparent difference minus its mean optimism, its 95% CI and p value are the 2.5th and 97.5th percentiles and the two-sided tail proportion of the per-resample differences in C-index evaluated on the original cohort. Within node-positive patients, the N2-versus-N1 hazard ratio was estimated unadjusted, stratified by origin and in the full model, and the LNR and the N2-versus-N1 contrast were tested over each other; the hazard ratio that this study had 80% power to detect was calculated from the number of deaths among node-positive patients. The proportional-hazards assumption was assessed by scaled Schoenfeld residuals.

Analyses of the influence of nodal yield were exploratory: the primary tests were repeated with the examined-node count added to the model, with the baseline hazard additionally stratified by period of resection (2013–2016 versus 2017–2024; as a check, also with period as a covariate), with both the count and period stratification, in the two periods separately, and after the 2018 change in pathology staffing. The Spearman correlation between the LNR and the positive-node count among node-positive patients was calculated by period, with bootstrap percentile 95% CIs from 5000 resamples. Node-positive patients were cross-classified by the N1/N2 boundary and by LNR at 0.18, a published threshold [12] prespecified for this analysis, and the discordant groups were described.

The remaining secondary analyses were likewise exploratory and hypothesis-generating. No correction for multiplicity was applied, by design: these analyses were intended to examine whether the primary comparisons were robust to specification rather than to establish additional findings, and all of them are reported. LNR was dichotomized from 0.05 to 0.45 in 0.05 increments to examine whether the primary result depended on the threshold; all nine results are reported, and none was selected. Effect modification by tumor origin (excluding duodenal cases) was tested with interaction terms and expressed as ratios of hazard ratios. Sensitivity analyses repeated the primary tests under alternative cohort definitions, covariate sets, CA19-9 specifications (including multiple imputation by chained equations), treatment adjustment and censoring rules; they are listed in Table S1. Two-sided *p* < 0.05 was considered significant. Analyses used R 4.3.3 with the survival (version 3.5-8), rms (version 6.7-1) and mice (version 3.16.0) packages; the code is provided as File S1. The code was written with assistance from a generative-AI tool under the authors’ direction; the corresponding author executed it in full on the analysis dataset, checked the output against every table, and re-derived the spline, bootstrap-optimism and multiple-imputation results independently of File S1 with standard package functions (rms::lrtest, rms::anova, rms::validate, Hmisc::rcorr.cens, mice::pool), which reproduced the reported values to the stated precision. Alternative tests (Wald tests; rms::validate with origin as a covariate) gave slightly different values with the same ordering.

## 3. Results

### 3.1. Cohort

Of the 244 pancreatic resections, 177 were pancreatoduodenectomies; 26 were excluded for histology other than invasive periampullary adenocarcinoma, one for having no examined node and three for distant metastasis, leaving 147 patients (Figure S1 and Table 1). The tumor arose from the pancreatic head in 49 (33.3%), the distal bile duct in 63 (42.9%), the ampulla in 31 (21.1%) and the duodenum in 4 (2.7%). The median number of examined nodes was 17 (interquartile range 12–23), 10 in 2013–2016 and 19 in 2017–2024; 76.9% of the specimens contained at least 12 nodes and 65.3% at least 15. Sixteen of the 63 distal bile duct specimens had fewer than 12. Seventy-four patients (50.3%) were node-positive, 38 N1 and 36 N2. N2 disease was concentrated in pancreatic head cancer (21 of 49) and was uncommon in distal bile duct (8 of 63) and ampullary cancer (6 of 31). Node-positive disease was found in 9 of 34 patients resected in 2013–2016 and 65 of 113 resected in 2017–2024, and N2 disease in 15% and 27%. Preoperative CA19-9 was not measured in 10 patients (6.8%). Over a median follow-up of 69.0 months, there were 77 deaths and 90 recurrence-or-death events. The median overall survival was 40.9 months (95% confidence interval [CI] 31.2–91.7). By origin, it was 24.1 months (18.5–36.8) in pancreatic head and 55.6 months (35.0–not reached) in distal bile duct cancer and was not reached in ampullary (lower limit 45.4 months; 11 deaths) or duodenal cancer (one death).

### 3.2. Incremental Information of the Nodal Descriptors

**Figure 1:**
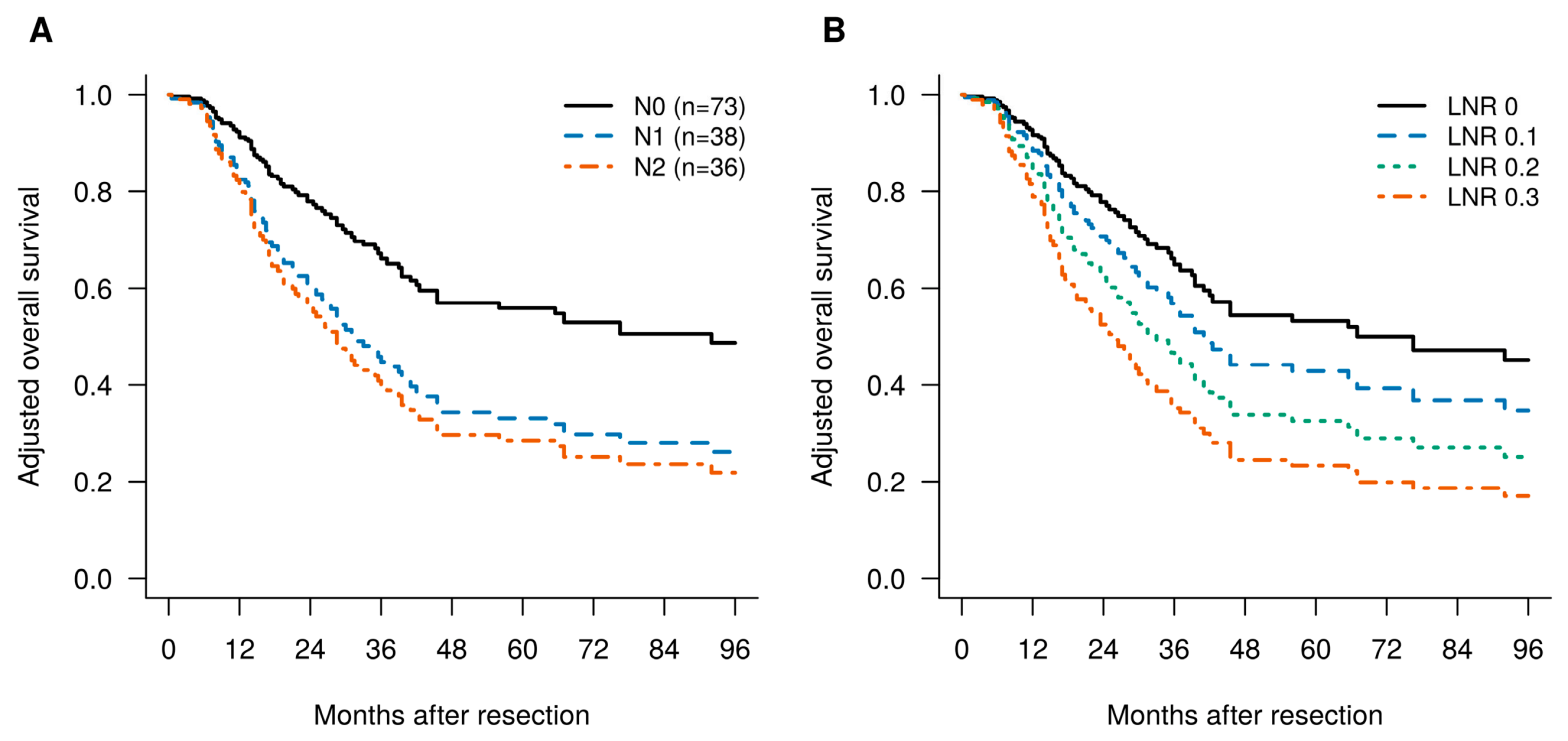
Adjusted overall survival after pancreatoduodenectomy by (**A**) AJCC 8th edition N category and (**B**) lymph node ratio (LNR) at values of 0, 0.1, 0.2 and 0.3 from the model with LNR as a continuous term. Curves are direct adjusted estimates from Cox models containing the clinicopathological covariates and stratified by tumor origin. The survival curve of each patient was predicted at each level of the descriptor, within the patient’s own origin stratum, and averaged over all 147 patients. Unadjusted Kaplan–Meier curves are shown in Figure S2.

In the multivariable model, the LNR was associated with a hazard ratio of 1.41 per 0.1 (95% CI 1.22–1.62, *p* < 0.001), and perineural invasion was the only other independent prognostic factor (Table 2). All five descriptors improved on the reference model (Table S2). Nodal positivity yielded a likelihood-ratio χ^2^ of 9.88 on 1 degree of freedom and the three-level N category 10.13 on 2, so the N1/N2 subdivision added χ^2^ 0.25 (*p* = 0.61; Table 3). Within the node-positive patients, the hazard ratio for N2 versus N1 was 1.53 (95% CI 0.87–2.67) unadjusted, 1.40 (0.75–2.63) stratified by origin and 1.21 (0.59–2.45) in the full model. Given 50 deaths among the 74 node-positive patients, a hazard ratio of about 2.2 would have been required for 80% power at a two-sided α of 0.05; the confidence interval is compatible with a clinically meaningful difference. N1 and N2 did not separate with an unadjusted Kaplan–Meier analysis (log-rank *p* = 0.13; Figure S2), and the adjusted survival curves of N1 and N2 lay close together (Figure 1A).

The LNR added information beyond positivity and beyond the three-level category, whereas the positive-node count did not. Added to nodal positivity, the LNR contributed χ^2^ 9.60 (*p* = 0.002; Figure 1B), whereas log(count + 1) contributed 1.00 (*p* = 0.32). Added to the three-level N category, the LNR contributed χ^2^ 10.01 (*p* = 0.002), and the N category did not add to the LNR (χ^2^ 1.25 on 2 degrees of freedom, *p* = 0.54). In the model containing positivity and the LNR, the N1/N2 subdivision contributed χ^2^ 0.66 (*p* = 0.42). For recurrence-free survival, the pattern was the same in the primary model, with the LNR adding to the N category (*p* = 0.020) and the N category adding to the LNR with *p* = 0.055 (Table S2; see Section 3.3 for period stratification). LODDS, which also incorporates the examined count, gave results similar to the LNR (Table S2). Within the node-positive patients, the LNR remained prognostic (hazard ratio 1.34 per 0.1) and added information beyond the N2-versus-N1 contrast (χ^2^ 7.94, *p* = 0.005), whereas the contrast did not add to the LNR (*p* = 0.59).

Although the spline point estimates suggested a steeper gradient at low ratios (Figure S3), the formal tests gave no evidence of departure from log-linearity for the LNR (p = 0.24 with three knots, 0.49 with four), and the spline gave the same conclusions as the linear term (LNR over the N category χ^2^ 13.19 on 2 degrees of freedom, *p* = 0.001; N category over LNR p = 0.22). For the positive-node count, the spline analyses suggested nonlinearity, particularly for recurrence-free survival (*p* = 0.060 for overall and 0.025 for recurrence-free survival), and log(count + 1) improved the model fit over the linear specification (ΔAIC −7.7 versus −3.7). The LNR added information to log(count + 1) (χ^2^ 9.33, *p* = 0.002) but not the reverse (*p* = 0.69). The optimism-corrected C-index was 0.649 for the reference model, 0.686 with nodal positivity, 0.683 with the N category and 0.703 with the LNR, a difference of 0.020 (95% CI 0.002–0.041; Table S3).

### 3.3. Influence of Nodal Yield

An adjustment for the examined-node count narrowed the difference between the descriptors. With the examined-node count in the model, that count carried a hazard ratio of 0.932 per node (95% CI 0.899–0.967) alongside the N category and 0.963 (0.935–0.992) alongside the LNR. In this model, the N1/N2 subdivision contributed χ^2^ 3.65 (*p* = 0.056) beyond positivity, the LNR contributed 7.03 (*p* = 0.008) beyond positivity and 4.03 (*p* = 0.045) beyond the N category, and the N category did not add to the LNR (*p* = 0.15). The subdivision added χ^2^ 0.64 (*p* = 0.42) beyond positivity plus the LNR. The interaction between the LNR and the examined-node count was not significant (*p* = 0.63).

Stratifying the baseline hazard by the period of resection, in addition to the origin, did not change the conclusions for overall survival: beyond positivity, the subdivision contributed χ^2^ 0.07 (*p* = 0.78) and the LNR, 4.85 (*p* = 0.028); the LNR added to the N category (χ^2^ 5.01, *p* = 0.025) whereas the N category did not add to the LNR (*p* = 0.39), although the LNR increment was about half that of the unstratified analysis (Table S1). The hazard ratio for the examined-node count alongside the N category was 0.940 per node (95% CI 0.906–0.976) with period stratification and 0.936 (0.902–0.972) with the period as a covariate. With both the examined-node count and period stratification in the model, the subdivision contributed χ^2^ 2.39 (*p* = 0.12) beyond positivity and the LNR, 1.57 (*p* = 0.21) beyond the N category. For recurrence-free survival, period stratification reversed the bidirectional comparison: the LNR did not add to the N category (χ^2^ 1.37, *p* = 0.24), whereas the N category added to the LNR (χ^2^ 9.64 on 2 degrees of freedom, *p* = 0.008), an increment that lay in the positivity step (χ^2^ 9.57, *p* = 0.002) rather than in the N1/N2 subdivision (*p* = 0.80).

The periods 2013–2016 and 2017–2024 differed in case mix (pancreatic head, 6 of 34 versus 43 of 113) and in the recorded frequency of perineural invasion (50% versus 79%) and lymphovascular invasion (32% versus 81%). In the 113 patients resected from 2017 onward (56 deaths; median yield 19), nodal positivity contributed χ^2^ 6.39, and beyond positivity, the subdivision contributed 0.49 (*p* = 0.49) and the LNR 1.90 (*p* = 0.17). The LNR did not add significantly to the N category (χ^2^ 1.49, *p* = 0.22), nor the N category to the LNR (*p* = 0.51). The LNR hazard ratio in this period, 1.34 (95% CI 1.08–1.67), did not differ from that before 2017 (interaction *p* = 0.96), and the LNR likewise did not add significantly to the N category from 2018 onward (*p* = 0.19). What changed was the relationship between the descriptors. Among the node-positive patients resected in 2013–2016, when the examined count ranged from 1 to 35, the LNR and the positive-node count were uncorrelated (Spearman ρ −0.02; bootstrap 95% CI −0.78 to 0.77, on nine patients). The four N1 patients of that period had a median of 3.5 examined nodes and a median LNR of 0.54. From 2017 onward, the LNR and the count were nearly collinear (ρ 0.90, 95% CI 0.81–0.94), the median LNR of N1 patients was 0.10 (interquartile range 0.05–0.14) and only 3 of 34 exceeded 0.2. Excluding the nine node-positive patients from 2013–2016 reduced the increment of the LNR over the N category in the whole cohort from χ^2^ 10.01 to 2.84 (*p* = 0.092). The increments in the subsets with at least 12 and at least 15 examined nodes (Table S1) likewise depended on the early node-positive patients they contained; without the six such patients in the subset with at least 15 nodes, the increment was χ^2^ 3.00 (*p* = 0.083). From 2018 onward, the N1/N2 subdivision itself added χ^2^ 3.35 (*p* = 0.067) beyond positivity, against 0.25 in the whole cohort. The nodal yield by year and the relationship between the LNR and the positive-node count by period are shown in Figure S4.

**Figure 2:**
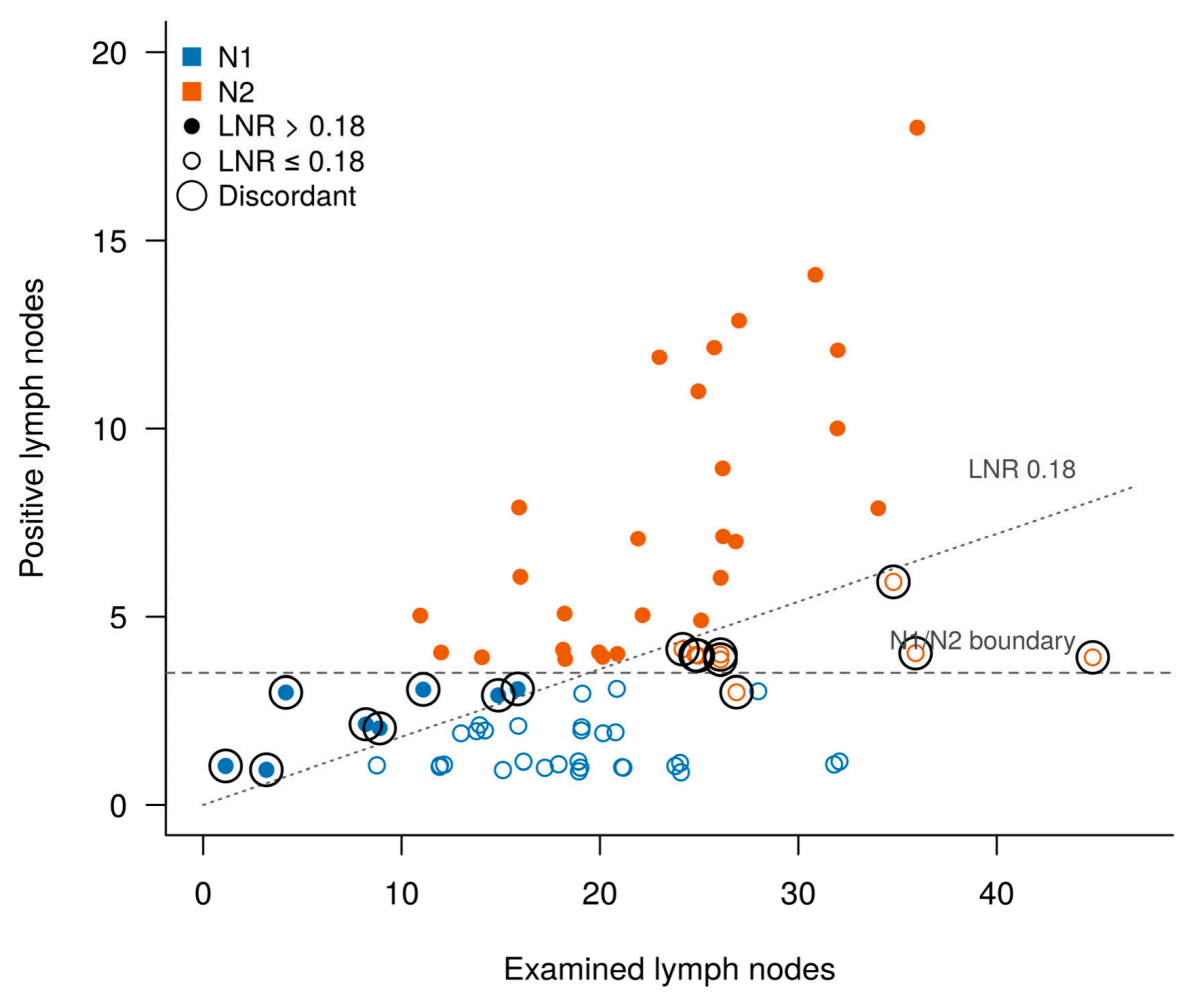
Discordance between the N category and the lymph node ratio among the 74 node-positive patients. Each point is one patient, plotted by examined and positive nodes. Color gives the AJCC 8th edition category; filled points have a ratio above 0.18 [12] and open points at or below it. The dashed line is the N1/N2 boundary of 1–3 versus ≥4 positive nodes (one duodenal patient with three positive nodes is N2 under the small-intestine definition), and the dotted line the ratio of 0.18. Ringed points are the 17 patients classified differently by the two descriptors (Table S4).

Cross-classifying the 74 node-positive patients by the N1/N2 boundary and by the LNR at 0.18 gave groups of similar size (38 and 36 versus 35 and 39) but reassigned 17 patients (Figure 2 and Table S4). The eight moved from N1 to the high-ratio group had one to three positive nodes among a median of 8.5 examined (interquartile range 3.75–12, range 1–16); seven died, with a median survival of 18.6 months. The nine moved from N2 to the low-ratio group had three to six positive nodes among a median of 26 examined (25–35, range 24–45); three died and median survival was not reached. The log-rank test between the two reassigned groups gave *p* = 0.013, on 10 deaths among 17 patients. Concordant N1 and N2 patients had medians of 19 and 23 examined nodes.

### 3.4. Exploratory and Sensitivity Analyses

The remaining analyses were exploratory. Dichotomized at each of nine thresholds from 0.05 to 0.45, the LNR carried adjusted hazard ratios of 2.00 to 3.69 (Table S5 and Figure S5). It added information beyond the N category at 0.15, 0.20 and 0.25 (*p* = 0.003, 0.001 and 0.021), with *p* = 0.057 at 0.10 and 0.059 at 0.30. The unadjusted LNR hazard ratio per 0.1 was 1.45 (95% CI 1.16–1.82) in pancreatic head, 1.42 (1.22–1.65) in distal bile duct and 1.65 (1.16–2.36) in ampullary cancer (Table S6). The ratios of hazard ratios relative to the pancreatic head were 1.10 (0.83–1.45) and 1.13 (0.76–1.70), and the interaction tests gave *p* = 0.76 for the LNR and 0.085 for the N category. The LNR added information beyond the N category in every sensitivity analysis except the two restricted to recent periods and the model containing both the examined-node count and period stratification, with hazard ratios per 0.1 of 1.34 to 1.57 (Table S1). The N category did not add to the LNR in any of these overall-survival analyses (*p* ≥ 0.089), and the subdivision added to positivity in none (*p* ≥ 0.056). Adding postoperative treatment to the model, as recorded, under a strict definition or as a time-dependent covariate, did not change these results. The proportional-hazards assumption was not violated in the primary models (global *p* = 0.97 and 0.93).

## 4. Discussion

In 147 periampullary adenocarcinomas, three findings emerged. First, most of the prognostic information in the AJCC 8th edition N category lay in nodal positivity: the N1/N2 subdivision added χ^2^ 0.25; its increment was larger, although still not significant, with the examined-node count in the model (χ^2^ 3.65) and from 2018 onward (3.35), and the estimate was imprecise. Second, the LNR added information beyond positivity and beyond the three-level category, for both endpoints in the primary models, with a modest gain in discrimination. Third, in the exploratory analyses, this increment appeared to depend on the nodal yield. It was attenuated after adjustment for the examined-node count, was about halved by stratification by period, and arose mainly from the node-positive patients resected before 2017, when the median yield was 10 and the examined count ranged from 1 to 35. It was not demonstrable in the 113 patients resected thereafter, among whom the ratio and the positive-node count were nearly collinear.

The subdivision entered the 8th edition [3] on the strength of series with medians of 24 and 19 examined nodes [4,5], and later validations asked whether N1 and N2 separate, not how much the separation adds. Where the increment has been quantified, it is small: a C-index gain of 0.003 to 0.02 [8,10,39], and a log-rank χ^2^ for N1 versus N2, one-third of that for N0 versus N1 in 2864 pancreatic head cancers [9]. Our estimate points the same way but is imprecise. The confidence interval for the adjusted N2-versus-N1 hazard ratio (0.59–2.45) includes the 1.3 to 1.4 implied by large series [10,25], and this study was powered only for a hazard ratio above 2.2. Two features of the cohort may also have limited the increment. First, the subdivision’s increment rose to χ^2^ 3.65 (*p* = 0.056) with the examined-node count in the model and to 3.35 (*p* = 0.067) from 2018 onward, suggesting that part of the information at the N1/N2 boundary is obscured by variable yield, although with period stratification added to the count-adjusted model, it was 2.39 (*p* = 0.12). Second, whether N1 and N2 separate in a given series appears to depend as much on the number of N2 patients as on the yield. Separation was reported with 593 N2 patients [10] and, narrowly, with 54 [25], but not with 12 to 17 [7,16,39]. Here, N2 disease was uncommon outside the pancreatic head, as in the Korean distal bile duct series [7,25,39]. The finding therefore concerns the size of the increment in a cohort of this composition, not whether the subdivision has value.

That the ratio predicts survival is not new [1,12,13,14]. The bidirectional test adds that, in this cohort, the LNR added information beyond the N category for overall survival, whereas no additional contribution of the N category beyond the LNR was detected; for recurrence-free survival, this held in the primary model but not once the baseline hazard was stratified by period, when nodal positivity added to the LNR and the LNR did not add to the N category. The relationship was adequately described by a linear term per 0.1 on the log-hazard scale, although the spline indicated a steeper gradient below 0.2, in keeping with the flattening of survival above that value reported elsewhere [40]. The positive-node count, by contrast, showed evidence of nonlinearity and, even when log-transformed, did not show a detectable increment over the N category. LODDS, which also incorporates the examined count, behaved similarly to the LNR [36]; it fitted marginally better and, similar to the LNR, lost much of its increment over the N category once the examined-node count was in the model (*p* = 0.24 and 0.045 for LODDS and LNR), but it is less familiar to clinicians, so the LNR is the focus of this report. The ratio’s improvement in model fit translated into a gain in C-index of 0.02, of the order expected from a single marker [41]; the data support the LNR as a source of additional prognostic information rather than of markedly better discrimination.

The third finding is exploratory and hypothesis-generating: the period analyses were added during revision, rest on nine early node-positive patients and are not supported by the formal interaction tests. It nevertheless qualifies the second finding. The reclassification analysis illustrates a plausible mechanism. The patients moved up by the ratio had few nodes examined and most died; the patients moved down had many nodes examined and most survived. A count cannot distinguish two positive nodes among eight from four among twenty-six, but a ratio can. This pattern is consistent with stage migration related to variation in the nodal yield, which Pawlik et al. described within the strata of positive-node count [13]; here, it appears at the N1/N2 boundary. Its corollary is that the ratio’s advantage would be expected to shrink as the yield becomes higher and more uniform, and the period analysis was consistent with this expectation. When the examined count varied less, the ratio became nearly a constant multiple of the count, and neither descriptor added to the other. Adjustment for the examined-node count, and stratification of the baseline hazard by period, similarly narrowed the difference between the descriptors. The objection that the ratio depends on the number of nodes examined [4,23] and the objection that the count suffices once yield is adequate [4,28] are thus two descriptions of the same phenomenon. Strobel et al. observed that only studies with more than 20 examined nodes had favored the count [4], and Malleo et al. estimated that 28 nodes are needed to detect N2 disease reliably [42]. Most of the published series, including the one in which the 8th edition category was validated [6], fall below these values. The yield in this cohort rose from 10 to 19 after a second surgeon joined the department and operated jointly with the first, and specimens with fewer than 10 nodes became uncommon after a pathologist with a hepato-pancreato-biliary focus joined; these are temporal associations. A low yield may also reflect tumor or host factors that are themselves prognostic. These alternatives affect the count-based category at least as much as the ratio, and none alters the observation that the relative information carried by the LNR was greater when the nodal yield was more variable.

The exploratory analyses are consistent with this reading. Every threshold between 0.15 and 0.25 added information beyond the N category in the whole cohort. The scan was not used to select a cutpoint, which in-sample optimization would have favored [26]; all nine results are reported, they are nested cuts of the same 74 patients, and we do not propose a threshold. The continuous ratio is the more natural descriptor. The LNR effect did not differ detectably between pancreatic head, distal bile duct and ampullary cancer. The ratios of hazard ratios had wide intervals, however, and the ampullary estimate rests on 11 deaths, so homogeneity is compatible with, not established by, these data. Distal bile duct cancer, in which comparisons of the ratio with the count have conflicted [5,16,17], made up 43% of the cohort, and the ratio was prognostic within it.

This study has limitations. The cohort is from a single center, accrued at about 13 resections per year, and is small; the events per parameter were seven to ten, and the models were used for comparative inference rather than individual prediction. The tests of the N1/N2 subdivision, of effect modification and of the N category over the LNR were underpowered, and their non-significance is not evidence of equivalence. The dependence of the LNR increment on the period rests on nine node-positive patients from 2013–2016, in whom the correlation between the LNR and the positive-node count (ρ −0.02) had a 95% CI of −0.78 to 0.77, and the point estimates from this subgroup are fragile. The formal interaction tests were uninformative, and the period effects other than the nodal yield, such as the shift in case mix toward pancreatic head cancer and changes in treatment or in pathological reporting (the recorded frequency of perineural and lymphovascular invasion also rose), cannot be excluded, although stratifying the baseline hazard by the period did not change the conclusions for overall survival. Stratification by origin does not remove the case-mix differences within the period subsets. The period change coincided with the changes in surgical and pathology staffing, whose contributions cannot be separated. The nodal stations were not recorded, so whether the anatomic distribution of the positive nodes differed between the periods could not be examined; station-level recording is a target for prospective data collection. The pT categories are defined differently across origins and were recorded under the AJCC edition in use at the time. The pT was an adjustment variable only, its coefficient should not be interpreted, and its omission did not change the results. The CA19-9 was measured at diagnosis without a concurrent bilirubin measurement, so cholestatic elevation could not be excluded; the results did not depend on how it was handled. Postoperative treatment was decided after pathological staging on the basis of tumor characteristics and patient condition and was given more often to node-positive patients; it was therefore excluded from the primary models, and its inclusion did not change the nodal comparisons. Duodenal adenocarcinoma contributed four patients, recurrence was analyzed only as a composite endpoint, and there is no external validation cohort.

## 5. Conclusions

In periampullary adenocarcinoma, the N1/N2 subdivision of the AJCC 8th edition added little prognostic information to nodal positivity, although the estimate was imprecise and the increment rose when the examined-node count was in the model. The lymph node ratio added information beyond the N category in the cohort as a whole, but the increment appeared to arise mainly from patients resected when the nodal yield was low and variable. When the yield was higher and more uniform, the ratio and the positive-node count conveyed nearly the same information. Since the LNR can be calculated from routinely reported node counts, it may provide complementary prognostic information when the nodal yield is low or variable; where the yield is consistently high, its additional value beyond the N category was not demonstrated and should not be assumed.

## Figures and Tables

**Table 1:** Clinicopathological characteristics and outcomes of the 147 patients by tumor origin.

Variable	Overall (n = 147)	Pancreatic Head (n = 49)	Distal Bile Duct (n = 63)	Ampulla of Vater (n = 31)	Duodenum (n = 4)	*p*
Age, years, median (IQR)	68 (61–73)	67 (60–74)	69 (65–75)	66 (60–72)	61 (54–67)	0.273
Male sex	83 (56.5)	24 (49.0)	41 (65.1)	16 (51.6)	2 (50.0)	0.324
Body-mass index, kg/m^2^, median (IQR)	23.7 (21.7–25.7)	23.6 (21.7–25.5)	24.1 (22.0–25.7)	23.2 (21.3–25.9)	20.6 (20.3–21.5)	0.184
ASA class ≥3	27 (18.4)	6 (12.2)	15 (23.8)	6 (19.4)	0 (0.0)	0.412
CA19-9 > 200 U/mL	47 (34.3)	23 (48.9)	16 (27.6)	8 (27.6)	0 (0.0)	0.058
Tumor size, cm, median (IQR)	2.5 (2.0–3.5)	3.0 (2.4–3.9)	2.6 (2.0–3.4)	2.0 (1.6–2.5)	3.2 (2.5–4.5)	0.001
pT3–4	44 (29.9)	13 (26.5)	15 (23.8)	14 (45.2)	2 (50.0)	0.112
Poorly differentiated or undifferentiated	19 (12.9)	6 (12.2)	10 (15.9)	3 (9.7)	0 (0.0)	0.872
R1 resection	16 (10.9)	8 (16.3)	8 (12.7)	0 (0.0)	0 (0.0)	0.086
Perineural invasion	106 (72.1)	43 (87.8)	47 (74.6)	15 (48.4)	1 (25.0)	<0.001
Lymphovascular invasion	102 (69.4)	40 (81.6)	43 (68.3)	18 (58.1)	1 (25.0)	0.026
Postoperative treatment	106 (72.1)	47 (95.9)	43 (68.3)	15 (48.4)	1 (25.0)	<0.001
Examined lymph nodes, median (IQR)	17 (12–23)	20 (15–26)	17 (11–21)	16 (12–20)	11 (9–16)	0.014
≥12 examined nodes	113 (76.9)	41 (83.7)	47 (74.6)	23 (74.2)	2 (50.0)	0.311
Node-positive	74 (50.3)	33 (67.3)	28 (44.4)	12 (38.7)	1 (25.0)	0.020
AJCC 8th edition N0/N1/N2	73/38/36	16/12/21	35/20/8	19/6/6	3/0/1	0.005
Lymph node ratio, median (IQR)	0.031 (0.000–0.163)	0.107 (0.000–0.222)	0.000 (0.000–0.098)	0.000 (0.000–0.189)	0.000 (0.000–0.028)	0.027
Deaths	77 (52.4)	34 (69.4)	31 (49.2)	11 (35.5)	1 (25.0)	0.010
Median overall survival, months (95% CI)	40.9 (31.2–91.7)	24.1 (18.5–36.8)	55.6 (35.0–NR)	NR (45.4–NR)	NR (16.8–NR)	

Values are n (%) or median (interquartile range, IQR). *p* values compare the four tumor origins by the Kruskal–Wallis test for continuous variables and Fisher’s exact test for categorical variables; for the N category, the test is a single test of the N0/N1/N2 distribution across the four origins. CA19-9 percentages are of the 137 patients in whom it was measured. Postoperative treatment (chemotherapy with or without radiotherapy, or radiotherapy alone) is descriptive and was not a covariate in the primary models. Medians of overall survival are Kaplan–Meier estimates; NR, not reached; the ampullary and duodenal estimates rest on 11 deaths and one death. ASA, American Society of Anesthesiologists; CA19-9, carbohydrate antigen 19-9; CI, confidence interval.

**Table 2:** Univariable and multivariable Cox models for overall survival.

Variable	Univariable HR (95% CI)	*p*	Multivariable HR (95% CI)	*p*
Age, per year	1.02 (0.99–1.05)	0.115	1.02 (0.99–1.05)	0.165
Male sex	1.01 (0.64–1.59)	0.974	1.03 (0.64–1.67)	0.909
pT3–4	1.85 (1.16–2.96)	0.010	1.09 (0.62–1.93)	0.768
Poorly differentiated or undifferentiated	2.41 (1.35–4.31)	0.003	1.73 (0.94–3.18)	0.077
R1 resection	1.66 (0.88–3.14)	0.119	1.33 (0.66–2.69)	0.423
Perineural invasion	2.72 (1.38–5.37)	0.004	2.16 (1.07–4.38)	0.032
Lymphovascular invasion	2.76 (1.49–5.13)	0.001	—	
CA19-9 > 200 U/mL	1.07 (0.66–1.74)	0.775	1.09 (0.65–1.85)	0.739
Node-positive (vs node-negative)	2.83 (1.72–4.67)	<0.001	—	
Lymph node ratio, per 0.1	1.45 (1.29–1.63)	<0.001	1.41 (1.22–1.62)	<0.001

Cox proportional hazards models with the baseline hazard stratified by tumor origin. Univariable models contain one variable each. The multivariable model is the reference model (age, sex, pT, differentiation, R1 status, perineural invasion and CA19-9, with a separate category for CA19-9 not measured) plus the lymph node ratio; nodal positivity and lymphovascular invasion were not entered with the ratio. Postoperative treatment was not a covariate (Methods). CA19-9, carbohydrate antigen 19-9; CI, confidence interval; HR, hazard ratio.

**Table 3:** Incremental prognostic information beyond nodal positivity for overall survival: nested likelihood-ratio tests.

Refinement	Added to Nodal Positivity: χ^2^ (df), *p*	Added to Nodal Positivity + N1/N2 Subdivision (the N Category): χ^2^ (df), *p*	N1/N2 Subdivision Added to Nodal Positivity + Refinement: χ^2^ (df), *p*
N1/N2 subdivision	0.25 (1), 0.614	—	—
Lymph node ratio, per 0.1	9.60 (1), 0.002	10.01 (1), 0.002	0.66 (1), 0.415
log(positive nodes + 1)	1.00 (1), 0.317	1.21 (1), 0.272	0.46 (1), 0.498

Nested likelihood-ratio tests within the reference model stratified by tumor origin. Nodal positivity itself contributed χ^2^ 9.88 (1 df) and the three-level N category 10.13 (2 df) when added to the reference model. The second column tests each refinement beyond nodal positivity; the third tests the lymph node ratio or the positive-node count beyond the full N category; the fourth tests whether the N1/N2 subdivision still adds information once nodal positivity and the refinement are both in the model. Results for recurrence-free survival, the log odds of positive nodes, the change in Akaike information criterion and the conventional two-degree-of-freedom reverse comparison are given in Table S2. df, degrees of freedom.

## Data Availability

The data presented in this study are available on request from the corresponding author, with consideration to the ethical restrictions on the patient-level data imposed by the Institutional Review Board. The analysis code is provided as File S1.

## Supplementary Materials

The following supporting information can be downloaded at: https://www.mdpi.com/article/10.3390/cancers18203321/s1. Figure S1: Patient flow; Figure S2: Unadjusted Kaplan–Meier estimates of overall survival by N category and lymph node ratio; Figure S3: Restricted cubic spline for the lymph node ratio; Figure S4: Nodal yield by year and the relationship between the lymph node ratio and the positive-node count by period; Figure S5: Forest plot of the lymph node ratio dichotomized at thresholds from 0.05 to 0.45; Table S1: Sensitivity analyses: the primary nested tests under alternative cohort definitions and model specifications (overall survival); Table S2: Model fit and nested likelihood-ratio tests for all nodal descriptors and both endpoints; Table S3: Optimism-corrected C-indices for overall survival and paired differences versus the model containing the AJCC 8th edition N category; Table S4: The 17 node-positive patients classified discordantly by the N1/N2 boundary and the lymph node ratio at 0.18; Table S5: Lymph node ratio dichotomized at thresholds from 0.05 to 0.45 (exploratory); Table S6: Lymph node ratio and overall survival by tumor origin, and tests of effect modification; File S1: Analysis code (R).

## Abbreviations

The following abbreviations are used in this manuscript:

AIC	Akaike information criterion
AJCC	American Joint Committee on Cancer
ASA	American Society of Anesthesiologists
CA19-9	Carbohydrate antigen 19-9
CI	Confidence interval
df	Degrees of freedom
HR	Hazard ratio
IQR	Interquartile range
LNR	Lymph node ratio
LODDS	Log odds of positive nodes
NR	Not reached
OS	Overall survival
REMARK	Reporting Recommendations for Tumor Marker Prognostic Studies
RFS	Recurrence-free survival
STROBE	Strengthening the Reporting of Observational Studies in Epidemiology
