# Supplementary material for: Incremental Prognostic Information Beyond Nodal Positivity: The AJCC 8th Edition N1/N2 Subdivision and the Lymph Node Ratio in Resected Periampullary Adenocarcinoma
Source: Cancers (Basel). 2026 Oct 15;18(20):3321. doi: 10.3390/cancers18203321 (PMC13702507; doi:10.3390/cancers18203321)
Supplement: Supplementary file 1 [file cancers-18-03321-s001.zip › cancers-4603896-supplementary.pdf]

cancers

MDPI

Supplementary Materials: Incremental Prognostic Information Beyond Nodal Positivity: The AJCC 8th-Edition N1/N2 Subdivision and the Lymph Node Ratio in Resected Periampullary Adenocarcinoma

Gun Hyung Na and Changho Seo *

Department of Surgery, Bucheon St. Mary's Hospital, College of Medicine, The Catholic University of Korea, 327 Sosa-ro, Wonmi-gu, Bucheon 14647, Republic of Korea; nagh0214@catholic.ac.kr
* Correspondence: schjee@catholic.ac.kr

Supplementary Figures

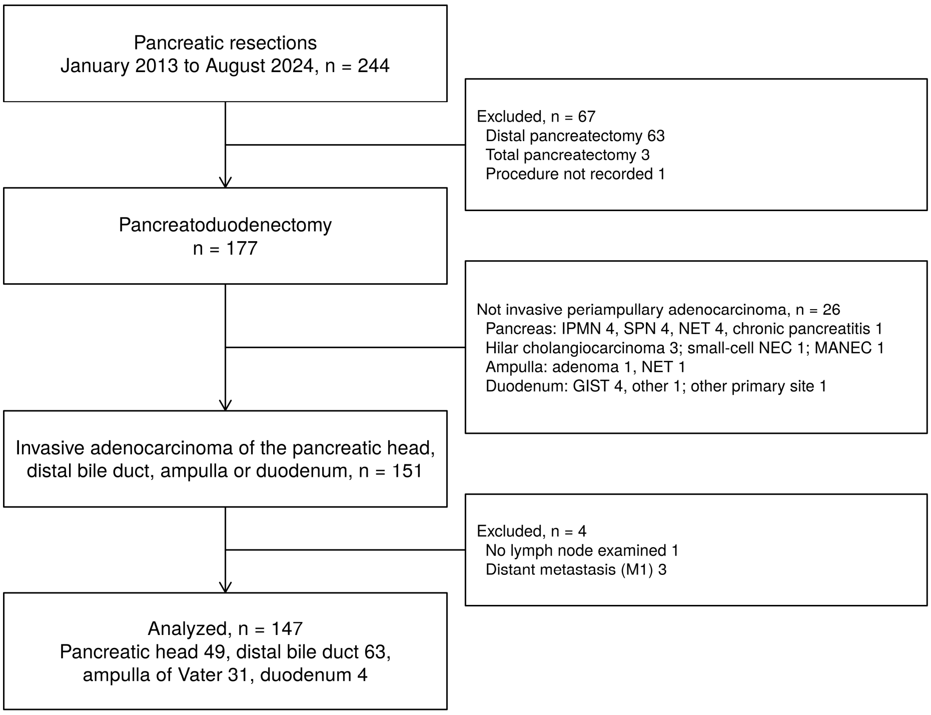

Figure S1. Patient flow. IPMN, intraductal papillary mucinous neoplasm; SPN, solid pseudopapillary neoplasm; NET, neuroendocrine tumor; NEC, neuroendocrine carcinoma; MANEC, mixed adenoneuroendocrine carcinoma; GIST, gastrointestinal stromal tumor.

 https://doi.org/10.3390/cancers18203321

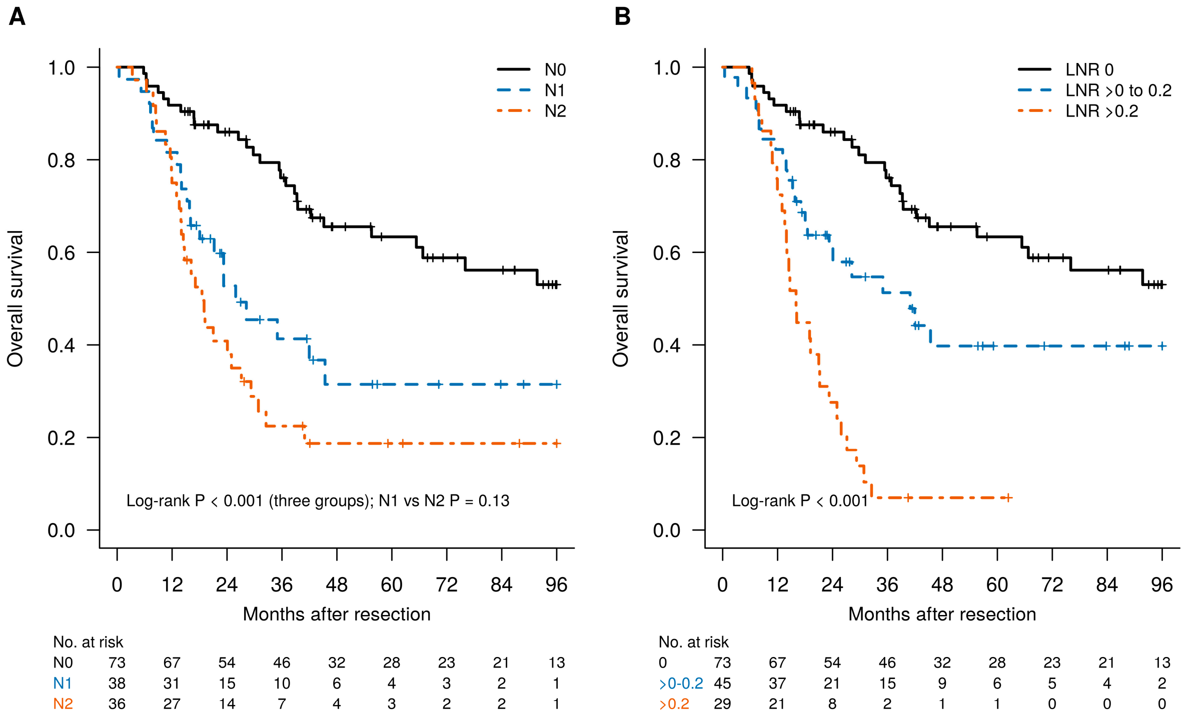

Figure S2. Unadjusted Kaplan–Meier estimates of overall survival by (**A**) AJCC 8th edition N category and (**B**) lymph node ratio (LNR; 0, >0 to 0.2, >0.2), with log-rank P values and numbers at risk.

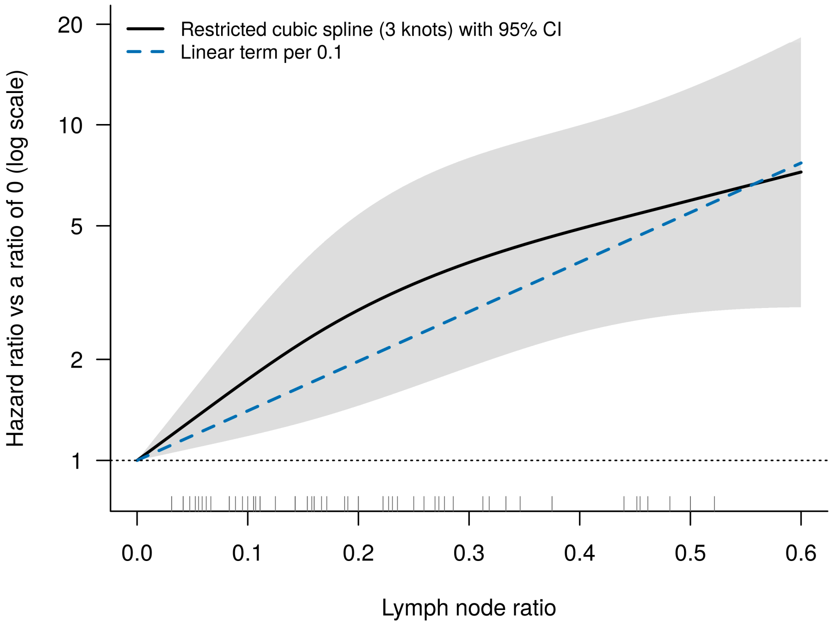

Figure S3. Hazard ratio for death by lymph node ratio relative to a ratio of 0, from the reference Cox model with the ratio entered as a restricted cubic spline with three knots (0.053, 0.163 and 0.454; solid line with 95% confidence band) or as a linear term per 0.1 (dashed line). The test of the nonlinear component gave P=0.24. The spline estimates hazard ratios of 1.74, 2.81 and 3.89 at ratios of 0.1, 0.2 and 0.3, against 1.41, 1.97 and 2.77 from the linear term.

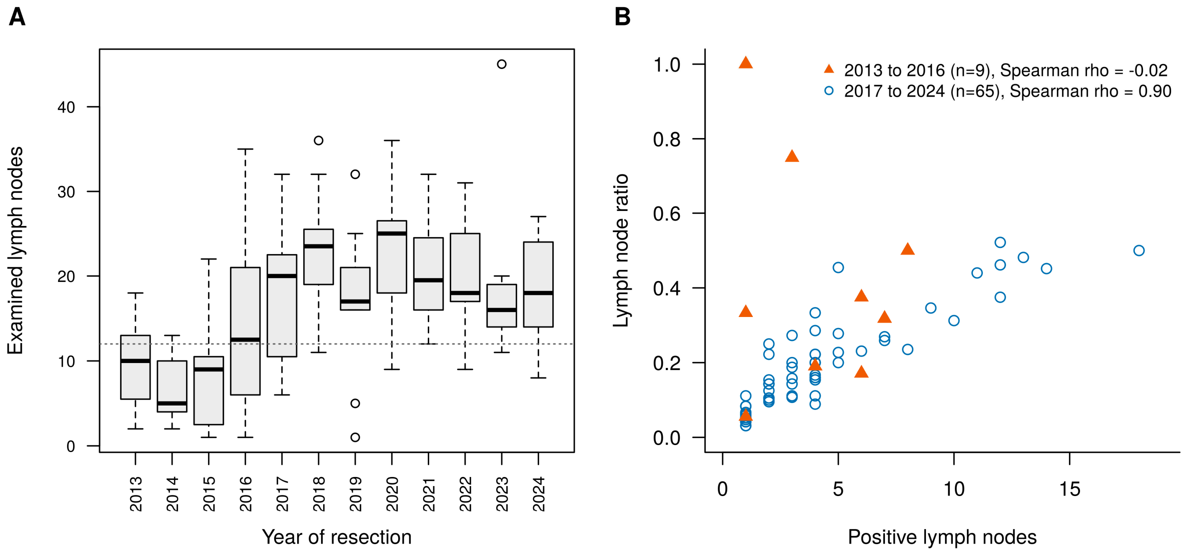

Figure S4. (**A**) Examined lymph nodes by year of resection (box plots; the line marks 12 nodes). (**B**) Lymph node ratio against the number of positive nodes among node-positive patients, by period of resection; the Spearman correlation was −0.02 (bootstrap 95% CI −0.78 to 0.77) in 2013–2016 and 0.90 (0.81–0.94) in 2017–2024.

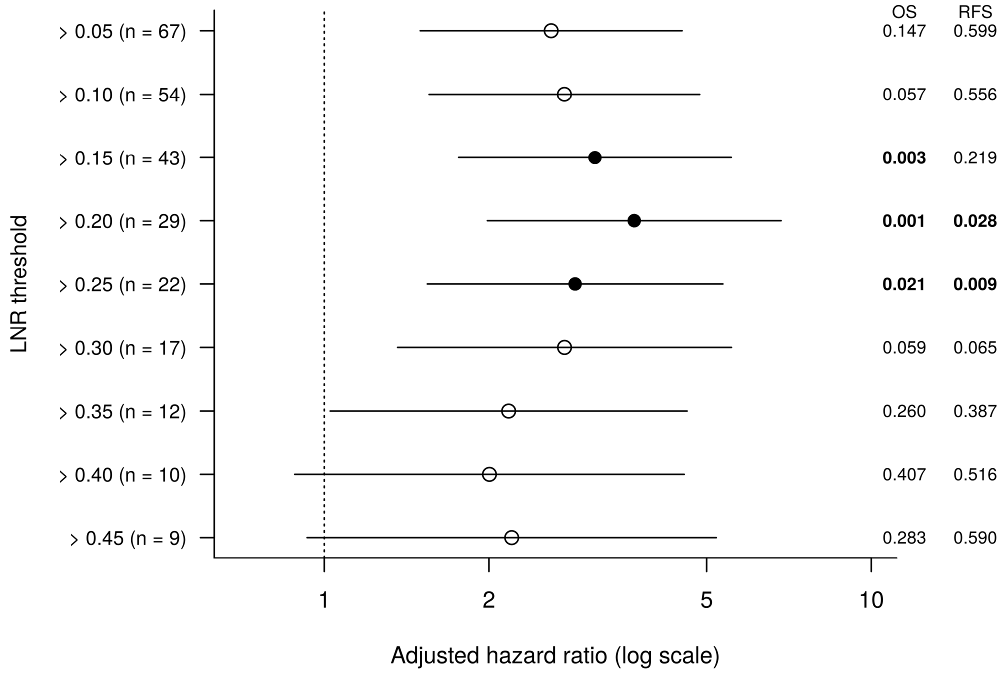

Figure S5. Lymph node ratio dichotomized at each of nine thresholds from 0.05 to 0.45 in steps of 0.05; n is the number of patients above each threshold. Points are adjusted hazard ratios for death (ratio above versus at or below the threshold) with 95% confidence intervals from the reference Cox model stratified by tumor origin; the horizontal axis is logarithmic. Filled points mark thresholds that added information to a model already containing the AJCC 8th edition N category for overall survival (nested likelihood-ratio test, P<0.05); the right-hand columns give that P value for overall (OS) and recurrence-free survival (RFS), in bold where P<0.05. These analyses are exploratory.

Supplementary Tables

Table S1. Sensitivity analyses: the primary nested tests under alternative cohort definitions and model specifications (overall survival).

Analysis	n	Deaths	LNR HR per 0.1 (95% CI)	N1/N2 subdivision added to positivity: χ^2, P	LNR added to N0/N1/N2: χ^2, P	N0/N1/N2 added to LNR: P	Subdivision added to positivity + LNR: P
**Cohort definition**							
Primary analysis	147	77	1.41 (1.22–1.62)	0.25, 0.614	10.01, 0.002	0.536	0.415
Excluding duodenal adenocarcinoma (n=143)	143	76	1.40 (1.22–1.62)	0.23, 0.635	9.98, 0.002	0.527	0.407
N1/N2 cut-point 1–3 / ≥4 applied to all origins	147	77	1.41 (1.22–1.62)	0.25, 0.614	10.01, 0.002	0.536	0.415
Excluding death within 90 days (n=146)	146	76	1.41 (1.22–1.63)	0.41, 0.525	10.23, 0.001	0.638	0.502
Examined nodes ≥12 (n=113)	113	58	1.54 (1.24–1.91)	1.83, 0.176	5.40, 0.020	0.455	0.491
Examined nodes ≥15 (n=96)	96	48	1.57 (1.25–1.98)	1.51, 0.219	5.98, 0.015	0.491	0.431
Resected 2017 onward (n=113)	113	56	1.34 (1.08–1.67)	0.49, 0.486	1.49, 0.223	0.512	0.783
Resected 2018 onward (n=102)	102	50	1.46 (1.14–1.87)	3.35, 0.067	1.73, 0.188	0.699	0.502
Upfront surgery only (n=142)	142	73	1.39 (1.20–1.61)	0.38, 0.540	8.86, 0.003	0.664	0.508
Excluding adenosquamous carcinoma (n=143)	143	73	1.42 (1.22–1.64)	0.29, 0.591	10.37, 0.001	0.603	0.452
Pancreatobiliary origins only (n=112)	112	65	1.40 (1.20–1.62)	0.42, 0.518	10.91, <0.001	0.904	0.655
**Model specification**							
Tumor origin as a covariate instead of stratum	147	77	1.39 (1.21–1.60)	0.28, 0.596	9.78, 0.002	0.525	0.383
Reduced covariate set (pT, PNI)	147	77	1.44 (1.26–1.64)	0.40, 0.527	13.16, <0.001	0.646	0.578
Lymphovascular invasion added	147	77	1.36 (1.17–1.59)	0.26, 0.613	9.73, 0.002	0.701	0.435
Calendar year of resection added	147	77	1.41 (1.23–1.62)	0.13, 0.723	7.81, 0.005	0.224	0.459
Examined-node count added	147	77	1.41 (1.23–1.61)	3.65, 0.056	4.03, 0.045	0.152	0.424
Baseline hazard stratified by period of resection	147	77	1.36 (1.17–1.59)	0.07, 0.785	5.01, 0.025	0.386	0.623
Examined-node count added, stratified by period	147	77	1.37 (1.18–1.60)	2.39, 0.122	1.57, 0.210	0.089	0.361
pT omitted	147	77	1.42 (1.24–1.61)	0.59, 0.443	11.76, <0.001	0.553	0.427
Origin-specific pT terms	147	77	1.43 (1.23–1.66)	0.26, 0.607	10.94, <0.001	0.471	0.326
Pancreatic head pT re-derived from tumor size (8th edition)	147	77	1.42 (1.22–1.64)	0.19, 0.665	9.97, 0.002	0.549	0.424
**CA19-9 handling**							
CA19-9 at 37 U/mL	147	77	1.40 (1.21–1.62)	0.44, 0.509	9.11, 0.003	0.560	0.481
CA19-9 at 100 U/mL	147	77	1.41 (1.22–1.62)	0.27, 0.603	9.85, 0.002	0.532	0.424
log CA19-9, continuous	147	77	1.41 (1.22–1.63)	0.25, 0.620	10.46, 0.001	0.514	0.348
CA19-9 omitted	147	77	1.42 (1.23–1.64)	0.57, 0.450	10.43, 0.001	0.633	0.531
Preoperative bilirubin >2 mg/dL added	147	77	1.42 (1.23–1.65)	0.07, 0.795	12.31, <0.001	0.315	0.157
CA19-9 multiply imputed (m=20; P values only)	147	77	1.42 (1.23–1.64)	0.458	<0.001	0.636	—

Analysis	n	Deaths	LNR HR per 0.1 (95% CI)	N1/N2 subdivision added to positivity: χ^2, P	LNR added to N0/N1/N2: χ^2, P	N0/N1/N2 added to LNR: P	Subdivision added to positivity + LNR: P
**Postoperative treatment and censoring**							
Postoperative treatment added, as recorded (originally submitted covariate set)	147	77	1.41 (1.22–1.62)	0.17, 0.677	9.75, 0.002	0.489	0.399
Postoperative treatment added (strict definition)	147	77	1.41 (1.22–1.63)	0.11, 0.739	9.10, 0.003	0.379	0.366
Postoperative treatment as a time-dependent covariate	144	74	1.40 (1.21–1.62)	0.21, 0.650	9.63, 0.002	0.713	0.493
Survivors censored at the last clinic visit (originally submitted rule)	147	77	1.44 (1.25–1.66)	0.25, 0.619	9.25, 0.002	0.274	0.454

All models are stratified by tumor origin and contain the reference covariates unless stated. CA19-9 was handled as in the primary analysis (>200 U/mL, with a category for not measured) unless stated; the alternative specifications use thresholds of 37 and 100 U/mL, a log-transformed continuous term with an indicator for not measured, omission, the addition of preoperative total bilirubin >2 mg/dL, and multiple imputation by chained equations (20 imputations, CA19-9 imputed on the log scale from the covariates, nodal variables and outcome). The strict definition of postoperative treatment requires a start within 180 days of resection and before any recurrence (88 patients; of the 106 recorded as treated, 18 did not meet it: all 18 started treatment on or after the recorded date of recurrence, nine of them also more than 180 days after resection); the time-dependent model enters treatment from its start date (three patients without a start date excluded). For multiple imputation, P values are from pooled Wald (D1) tests. With the baseline hazard stratified by period of resection (2013–2016 versus 2017–2024), the corresponding tests for recurrence-free survival gave: subdivision added to positivity χ^2 0.56 (P=0.45); LNR added to N0/N1/N2 χ^2 1.37 (P=0.24); N0/N1/N2 added to LNR χ^2 9.64 (P=0.008), of which nodal positivity added to LNR χ^2 9.57 (P=0.002) and the subdivision added to positivity plus LNR P=0.80. The uniform cut-point row is identical to the primary analysis because the only patient it reclassifies (duodenal, three positive nodes) was censored before the single death in the duodenal stratum and therefore does not enter the stratified partial likelihood. CA19-9, carbohydrate antigen 19-9; CI, confidence interval; HR, hazard ratio; LNR, lymph node ratio; PNI, perineural invasion.

Table S2. Model fit and nested likelihood-ratio tests for all nodal descriptors and both endpoints.

Endpoint	Descriptor	ΔAIC	Added to the reference model: χ^2 (df), P	Added to nodal positivity: χ^2 (df), P	Added to N0/N1/N2: χ^2 (df), P	N0/N1/N2 added to it: χ^2 (df), P	N1/N2 subdivision added to positivity + descriptor: χ^2 (df), P
Overall survival	Nodal positivity (AJCC 7th edition)	−7.9	9.88 (1), 0.002	—	—	0.25 (1), 0.614	—
	N0/N1/N2 (AJCC 8th edition)	−6.1	10.13 (2), 0.006	0.25 (1), 0.614	—	—	—
	log(positive nodes + 1)	−7.7	9.72 (1), 0.002	1.00 (1), 0.317	1.21 (1), 0.272	1.62 (2), 0.446	0.46 (1), 0.498

https://doi.org/10.3390/cancers18203321

**Endpoint**	**Descriptor**	**ΔAIC**	**Added to the reference model: χ^2 (df), P**	**Added to nodal positivity: χ^2 (df), P**	**Added to N0/N1/N2: χ^2 (df), P**	**N0/N1/N2 added to it: χ^2 (df), P**	**N1/N2 subdivision added to positivity + descriptor: χ^2 (df), P**
	Lymph node ratio, per 0.1	−16.9	18.90 (1), <0.001	9.60 (1), 0.002	10.01 (1), 0.002	1.25 (2), 0.536	0.66 (1), 0.415
	Log odds of positive nodes	−19.4	21.41 (1), <0.001	11.53 (1), <0.001	12.56 (1), <0.001	1.28 (2), 0.527	1.28 (1), 0.258
Recurrence-free survival	Nodal positivity (AJCC 7th edition)	−20.5	22.47 (1), <0.001	—	—	0.38 (1), 0.537	—
	N0/N1/N2 (AJCC 8th edition)	−18.8	22.85 (2), <0.001	0.38 (1), 0.537	—	—	—
	log(positive nodes + 1)	−18.7	20.67 (1), <0.001	1.51 (1), 0.219	1.69 (1), 0.194	3.87 (2), 0.145	0.56 (1), 0.454
	Lymph node ratio, per 0.1	−20.5	22.45 (1), <0.001	5.62 (1), 0.018	5.41 (1), 0.020	5.80 (2), 0.055	0.17 (1), 0.680
	Log odds of positive nodes	−22.0	24.01 (1), <0.001	4.96 (1), 0.026	4.76 (1), 0.029	3.60 (2), 0.166	0.18 (1), 0.671

Nested likelihood-ratio tests within the reference model stratified by tumor origin. ΔAIC is the change in Akaike information criterion when the descriptor is added to the reference model (negative values favor the larger model; the criterion penalizes each parameter by two units, so the two-parameter N category is penalized by two units more than the one-parameter descriptors; the comparison is descriptive). For the N0/N1/N2 row, "added to nodal positivity" is the increment of the N1/N2 subdivision. For the other descriptors, the last column tests whether the N1/N2 subdivision adds information to a model that already contains nodal positivity and the descriptor; "N0/N1/N2 added to it" is the conventional two-degree-of-freedom reverse test. The positive-node count is entered as log(count + 1) because spline analysis suggested nonlinearity; entered linearly it gave ΔAIC −3.7 and −11.9 for the two endpoints. AIC, Akaike information criterion; AJCC, American Joint Committee on Cancer; df, degrees of freedom.

Table S3. Optimism-corrected C-indices for overall survival and paired differences versus the model containing the AJCC 8th edition N category.

**Model**	**Apparent C-index**	**Optimism**	**Optimism-corrected C-index**	**Difference vs AJCC 8th N (95% CI)**	**P**
Clinicopathological model only	0.683	0.034	0.649	−0.035 (−0.069 to −0.012)	0.006
+ Nodal positivity	0.719	0.033	0.686	+0.003 (−0.008 to +0.015)	0.974
+ N0/N1/N2 (AJCC 8th)	0.721	0.037	0.683	— (reference)	—
+ log(positive nodes + 1)	0.719	0.034	0.685	+0.002 (−0.010 to +0.019)	0.688
+ Lymph node ratio	0.733	0.029	0.703	+0.020 (+0.002 to +0.041)	0.034
+ Log odds of positive nodes	0.736	0.028	0.709	+0.025 (+0.010 to +0.045)	0.002

Harrell's C-index with bootstrap optimism correction (1000 resamples): each model was refitted on each resample, the optimism is the mean difference between the C-index of the refitted model on the resample and on the original cohort, and the corrected C-index is the apparent C-index minus the optimism. Differences versus the N-category model are optimism-corrected in the same way; their 95% confidence intervals are percentile intervals of the paired per-resample differences in the C-index of the refitted models on the original cohort, and P values are twice the smaller tail proportion of those differences. Apparent, optimism and corrected values are rounded independently. All models are stratified by tumor origin and contain the reference covariates; the C-

index was computed from the linear predictor pooled across origin strata. The C-index is reported for context; the comparisons of the manuscript rest on the likelihood-ratio tests. AJCC, American Joint Committee on Cancer; CI, confidence interval.

Table S4. The 17 node-positive patients classified discordantly by the N1/N2 boundary and the lymph node ratio at 0.18.

Reclassification	Tumor origin	Examined nodes	Positive nodes	LNR	Year	Status	Follow-up, months
N1 → LNR > 0.18	Distal bile duct	1	1	1.00	2016	Died	7.0
N1 → LNR > 0.18	Ampulla	3	1	0.33	2015	Died	25.9
N1 → LNR > 0.18	Distal bile duct	4	3	0.75	2014	Died	21.2
N1 → LNR > 0.18	Pancreatic head	8	2	0.25	2024	Died	10.8
N1 → LNR > 0.18	Distal bile duct	9	2	0.22	2017	Died	16.0
N1 → LNR > 0.18	Pancreatic head	11	3	0.27	2023	Died	23.3
N1 → LNR > 0.18	Ampulla	15	3	0.20	2018	Died	8.0
N1 → LNR > 0.18	Ampulla	16	3	0.19	2023	Alive	27.0
N2 → LNR ≤ 0.18	Distal bile duct	24	4	0.17	2021	Died	40.9
N2 → LNR ≤ 0.18	Pancreatic head	25	4	0.16	2022	Died	17.1
N2 → LNR ≤ 0.18	Pancreatic head	25	4	0.16	2022	Alive	42.1
N2 → LNR ≤ 0.18	Pancreatic head	26	4	0.15	2022	Died	11.6
N2 → LNR ≤ 0.18	Pancreatic head	26	4	0.15	2020	Alive	59.1
N2 → LNR ≤ 0.18	Duodenum	27	3	0.11	2024	Alive	15.2
N2 → LNR ≤ 0.18	Pancreatic head	35	6	0.17	2016	Alive	114.4
N2 → LNR ≤ 0.18	Pancreatic head	36	4	0.11	2018	Alive	87.9
N2 → LNR ≤ 0.18	Distal bile duct	45	4	0.09	2023	Alive	27.7

Patients are ordered by direction of reclassification and number of examined nodes. The duodenal patient with three positive nodes is N2 under the small-intestine definition of the AJCC 8th edition. Concordant N1 (n=30) and N2 (n=27) patients had medians of 19 (range 9–32) and 23 (11–36) examined nodes. LNR, lymph node ratio.

Table S5. Lymph node ratio dichotomized at thresholds from 0.05 to 0.45 (exploratory).

Threshold	n above	Adjusted HR (95% CI)	P	Added to N0/N1/N2, OS: χ^2 (1 df), P	Added to N0/N1/N2, RFS: P
> 0.05	67	2.60 (1.50–4.51)	<0.001	2.11, 0.147	0.599
> 0.10	54	2.75 (1.55–4.86)	<0.001	3.62, 0.057	0.556
> 0.15	43	3.12 (1.76–5.55)	<0.001	8.79, 0.003	0.219
> 0.20	29	3.69 (1.99–6.84)	<0.001	10.28, 0.001	0.028
> 0.25	22	2.87 (1.54–5.35)	<0.001	5.31, 0.021	0.009
> 0.30	17	2.75 (1.36–5.55)	0.005	3.56, 0.059	0.065
> 0.35	12	2.17 (1.03–4.61)	0.043	1.27, 0.260	0.387
> 0.40	10	2.00 (0.88–4.55)	0.096	0.69, 0.407	0.516
> 0.45	9	2.20 (0.93–5.21)	0.073	1.15, 0.283	0.590

Each dichotomy was entered in the reference model stratified by tumor origin and then tested for incremental information over the AJCC 8th edition N category. The nine dichotomies are nested cuts of the same 74 node-positive patients; no threshold was selected. CI, confidence interval; HR, hazard ratio; OS, overall survival; RFS, recurrence-free survival.

Table S6. Lymph node ratio and overall survival by tumor origin, and tests of effect modification.

Tumor origin	n	Deaths	Node-positive	Unadjusted HR per 0.1 (95% CI)	P
Pancreatic head	49	34	33	1.45 (1.16–1.82)	0.001

Tumor origin	n	Deaths	Node-positive	Unadjusted HR per 0.1 (95% CI)	P
Distal bile duct	63	31	28	1.42 (1.22–1.65)	<0.001
Ampulla	31	11	12	1.65 (1.16–2.36)	0.006
Ratio of hazard ratios vs pancreatic head				Distal bile duct 1.10 (0.83–1.45); ampulla 1.13 (0.76–1.70)	
Interaction with origin (likelihood-ratio test)				LNR P=0.76; LODDS P=0.71; N category P=0.085	

Hazard ratios are from unadjusted Cox models within each origin. Interaction terms were tested in the covariate-adjusted model with origin as a covariate, excluding the four duodenal cases; ratios of hazard ratios are the exponentiated interaction coefficients. The ampullary estimate rests on 11 deaths. CI, confidence interval; HR, hazard ratio; LNR, lymph node ratio; LODDS, log odds of positive nodes.

File S1

File S1 (File_S1_analysis_code.R) contains the complete R code that reproduces every statistic and table of the manuscript and of these Supplementary Materials, and the estimates on which the figures are based, from the analysis dataset. The variables it requires are listed in its header. The script is reproduced in full below; a plain-text copy is also provided.

```
##################################################################################
## File S1. Analysis code (second revised version, October 2026)
##
## Na GH, Seo C. Incremental prognostic information beyond nodal positivity:
## the AJCC 8th-edition N1/N2 subdivision and the lymph node ratio in resected
## periampullary adenocarcinoma. Cancers (manuscript cancers-4603896, revision 2).
##
## This script reproduces every statistic and table of the revised manuscript and
## Supplementary Materials, and the estimates on which the figures are based, from the
## analysis dataset (figure drawing code is available from the corresponding author). Patient-level
## data are not publicly deposited (see the Data Availability Statement).
##
## Required variables in `a` (one row per patient):
##   os_time, os_event     overall survival (months from resection; death from any cause).
##                         Survivors censored at the last date known alive (clinical or
##                         administrative records), follow-up closed 31 October 2025.
##   rfs_time, rfs_event   recurrence-free survival; event = recurrence or death; event-free
##                         survivors censored at the last clinical assessment.
##   os_time_visit         overall survival time with survivors censored at the last clinic
##                         visit (specification of the originally submitted manuscript).
##   death90               1 if death within 90 days of resection (kept in the primary cohort).
##   age, sexf, Tf, gradef, R1, pni, lvi, ca (<=200 / >200 U/mL / not measured), ca199, tbil
##   adj, adj_type, adj_start/adj_days, recur, recur_date   postoperative treatment (sensitivity only)
##   origin  factor: Pancreatic head / Distal bile duct / Ampulla / Duodenum
##   ln_total, ln_pos, lnr, lnr10 (per 0.1), lodds, npos
##   N7   N0 / N+ ;  N8  N0 / N1 / N2 by the organ-specific AJCC 8th-edition definition
##        (1-3 / >=4 for pancreatic head, distal bile duct and ampulla; 1-2 / >=3 for duodenum)
##   N8u  N0 / N1 / N2 with 1-3 / >=4 applied to all origins (sensitivity)
##   yr, neoadj, histo_txt, operator, size_x, ed (AJCC edition recorded in the report)
##   id, op_date, bmi, asa, pT, drain_yn, ca_days_before_op, tbil_days_before_op (descriptives)
##
## R 4.3.3; packages survival 3.5-8, rms, mice, jsonlite. Section labels follow the manuscript.
##################################################################################
suppressMessages({library(survival); library(splines); library(rms); library(mice); library(jsonlite)})
set.seed(20260905)
a <- readRDS("data/analysis_final.rds")
R <- list()                              # all numbers collected here and written to output/re-
sults_v2.json
pr <- function(...) cat(sprintf(...), "\n")


## Reference model: prespecified clinicopathological covariates, baseline hazard stratified by tumor origin.
## Postoperative treatment (chemotherapy +/- radiotherapy, or radiotherapy alone) is NOT in the reference
model (it is determined after staging; see Methods).
B   <- "age+sexf+Tf+gradef+R1+pni+ca+strata(origin)"
COV <- "age+sexf+Tf+gradef+R1+pni+ca"
f  <- function(rhs, d = a, ep = "os") coxph(as.formula(sprintf("Surv(%s_time,%s_event)~%s", ep, ep, rhs)),
data = d)
LR <- function(m1, m2) { an <- anova(m1, m2); c(chi = an$Chisq[2], df = an$Df[2], p = an$`Pr(>|Chi|)`[2]) }
hr <- function(m, term) { s <- summary(m)$conf.int; c(hr = s[term,1], lo = s[term,3], hi = s[term,4], p =
summary(m)$coef[term,5]) }
np <- droplevels(subset(a, N8 != "N0"))
kL <- quantile(np$lnr, c(.10,.50,.90)); SP <- paste0("rcs(lnr,c(", paste(kL, collapse=","), "))")   # 3-knot
restricted cubic spline for LNR
kC <- quantile(np$npos, c(.10,.50,.90)); SPC <- paste0("rcs(npos,c(", paste(kC, collapse=","), "))")
a$lognpos <- log(a$npos + 1)
```

```
cat("\n==================== 3.1 Cohort ====================\n")
R$cohort <- list(n = nrow(a), deaths = sum(a$os_event), rfs_events = sum(a$rfs_event),
  flow = c(screened = 244, PD = 177, adenocarcinoma = 151, nodes_examined = 150, M0 = 147, analyzed =
nrow(a)),
  origin = as.list(table(a$origin)), N8_by_origin = as.data.frame.matrix(table(a$origin, a$N8)),
  N8 = as.list(table(a$N8)), N7 = as.list(table(a$N7)),
  eln_median = median(a$ln_total), eln_q = unname(quantile(a$ln_total, c(.25,.75))), eln_ge12 = mean(a$ln_to-
tal >= 12), eln_ge15 = mean(a$ln_total >= 15),
  dcc_lt12 = sum(a$ln_total < 12 & a$origin == "Distal bile duct"), ca_missing = sum(is.na(a$ca199)),
  death90 = sum(a$death90), ampulla_deaths = sum(a$os_event[a$origin=="Ampulla"]), duodenum_deaths =
sum(a$os_event[a$origin=="Duodenum"]))
sf <- survfit(Surv(os_time, os_event) ~ 1, a); R$cohort$median_os <- unname(summary(sf)$table[c("me-
dian","0.95LCL","0.95UCL")])
R$cohort$median_fu <- unname(summary(survfit(Surv(os_time, 1-os_event) ~ 1, a))$table["median"])
R$cohort$median_fu_visit <- unname(summary(survfit(Surv(os_time_visit, 1-os_event) ~ 1, a))$table["median"])
sfo <- survfit(Surv(os_time, os_event) ~ origin, a); R$cohort$median_os_by_origin <- summary(sfo)$table[,
c("records","events","median","0.95LCL","0.95UCL")]
R$cohort$os5y_by_origin <- summary(sfo, times = 60, extend = TRUE)$surv
R$cohort$rfs_median <- unname(summary(survfit(Surv(rfs_time, rfs_event) ~ 1, a))$table[c("me-
dian","0.95LCL","0.95UCL")])
R$cohort$years <- as.numeric(diff(range(as.Date(a$op_date)))) / 365.25
print(R$cohort$N8_by_origin); pr("n %d, deaths %d, RFS events %d; ELN median %d (IQR %d-%d); >=12 %.1f%%;
median OS %.1f (%.1f-%.1f); median FU %.1f (last known alive) vs %.1f (last visit)",
    nrow(a), sum(a$os_event), sum(a$rfs_event), median(a$ln_total), quantile(a$ln_total,.25), quan-
tile(a$ln_total,.75), 100*mean(a$ln_total>=12), R$cohort$median_os[1], R$cohort$median_os[2], R$cohort$me-
dian_os[3], R$cohort$median_fu, R$cohort$median_fu_visit)
print(R$cohort$median_os_by_origin)


## Table 1 (characteristics by origin; Kruskal-Wallis for continuous, Fisher's exact for categorical; one
test per row)
t1 <- list(); fmt_med <- function(x, dg=1) sprintf(paste0("%.",dg,"f (%.",dg,"f-%.",dg,"f)"), me-
dian(x,na.rm=T), quantile(x,.25,na.rm=T), quantile(x,.75,na.rm=T))
fmt_np <- function(x) sprintf("%d (%.1f)", sum(x,na.rm=T), 100*mean(x,na.rm=T))
grp <- split(a, a$origin); row_c <- function(lab, v, dg=1) { x <- a[[v]]; c(lab, fmt_med(x,dg), sapply(grp,
function(g) fmt_med(g[[v]],dg)), sprintf("%.3f", kruskal.test(x ~ a$origin)$p.value)) }
row_b <- function(lab, x) { c(lab, fmt_np(x), sapply(split(x, a$origin), fmt_np), sprintf("%.3f",
fisher.test(table(a$origin, x))$p.value)) }
a$male <- a$sexf=="Male"; a$asa3 <- a$asa>=3; a$ca200 <- ifelse(is.na(a$ca199), NA, a$ca199>200); a$t34 <-
a$Tf=="pT3-4"; a$poor <- a$gradef!="well or moderate"; a$eln12 <- a$ln_total>=12; a$npos_b <- a$N7=="N+";
a$dead <- a$os_event==1
t1$rows <- rbind(row_c("Age, years", "age", 0), row_b("Male sex", a$male), row_c("Body-mass index, kg/m2",
"bmi"), row_b("ASA class >=3", a$asa3),
  c("CA19-9 > 200 U/mL (of measured)", fmt_np(a$ca200), sapply(split(a$ca200, a$origin), fmt_np),
sprintf("%.3f", fisher.test(table(a$origin[!is.na(a$ca200)], a$ca200[!is.na(a$ca200)]))$p.value)),
  row_c("Tumor size, cm", "size_x"), row_b("pT3-4", a$t34), row_b("Poorly differentiated or undifferenti-
ated", a$poor), row_b("R1 resection", a$R1==1), row_b("Perineural invasion", a$pni==1), row_b("Lymphovascular
invasion", a$lvi==1),
  row_b("Postoperative treatment", a$adj==1), row_c("Examined lymph nodes", "ln_total", 0), row_b(">=12 exam-
ined nodes", a$eln12), row_b("Node-positive", a$npos_b),
  c("N category (N0/N1/N2)", paste(table(a$N8), collapse="/"), sapply(grp, function(g) paste(table(g$N8),
collapse="/")), sprintf("%.3f", fisher.test(table(a$origin, a$N8))$p.value)),
  row_c("Lymph node ratio", "lnr", 3), row_b("Deaths", a$dead))
colnames(t1$rows) <- c("Variable", "Overall", levels(a$origin), "P"); R$table1 <- t1$rows; print(t1$rows,
quote = FALSE)
R$table1_N_fisher <- fisher.test(table(a$origin, a$N8))$p.value


## Nodal yield by period, surgeon (3.1 / 3.3)
a$period <- ifelse(a$yr >= 2017, "2017-2024", "2013-2016")
R$yield <- list(by_period = tapply(a$ln_total, a$period, function(x) c(n=length(x), median=median(x),
q1=quantile(x,.25), q3=quantile(x,.75), lt10=sum(x<10), lt12=sum(x<12))),
  by_year = tapply(a$ln_total, a$yr, median), lt10_three = c(`2013-2016`=sum(a$ln_total<10 & a$yr<2017),
`2017`=sum(a$ln_total<10 & a$yr==2017), `2018-2024`=sum(a$ln_total<10 & a$yr>=2018)),
  n_three = c(sum(a$yr<2017), sum(a$yr==2017), sum(a$yr>=2018)),
  by_surgeon_period = tapply(a$ln_total, list(a$operator, a$period), median), wilcox_period = wil-
cox.test(ln_total ~ period, a)$p.value,
  N2_by_period = tapply(a$N8=="N2", a$period, mean), Npos_by_period = tapply(a$N7=="N+", a$period, sum))
R$yield$n_by_surgeon <- table(a$operator); R$yield$origin_by_period <- table(a$period, a$origin);
R$yield$pni_lvi_by_period <- rbind(pni = tapply(a$pni, a$period, mean), lvi = tapply(a$lvi, a$period, mean))
print(R$yield$by_period); print(R$yield$lt10_three); print(R$yield$by_surgeon_period);
print(R$yield$n_by_surgeon); print(R$yield$origin_by_period); print(round(R$yield$pni_lvi_by_period, 2))


cat("\n==================== 3.2 Incremental information of the nodal descriptors ====================\n")
## Table 3 and Table S2 (symmetric structure): each refinement is tested beyond nodal positivity, and each
beyond the other
sym <- function(d = a, BB = B, ep = "os", N8v = "N8") {
  m0 <- f(BB, d, ep); m7 <- f(paste0(BB,"+N7"), d, ep); m8 <- f(paste0(BB,"+",N8v), d, ep)
  out <- list(positivity = c(LR(m0, m7), dAIC = AIC(m7)-AIC(m0)), N8 = c(LR(m0, m8), dAIC = AIC(m8)-AIC(m0)),
            subdiv_over_pos = LR(m7, m8))
  for (v in c("lnr10","lodds","lognpos","npos")) {
    mv <- f(paste0(BB,"+",v), d, ep)
    out[[v]] <- list(over_ref = c(LR(m0, mv), dAIC = AIC(mv)-AIC(m0)), over_pos = LR(m7,
f(paste0(BB,"+N7+",v), d, ep)),
                     over_N8 = LR(m8, f(paste0(BB,"+",N8v,"+",v), d, ep)), N8_over = LR(mv,
f(paste0(BB,"+",v,"+",N8v), d, ep)),
                     pos_over = LR(mv, f(paste0(BB,"+",v,"+N7"), d, ep)), subdiv_over_pos_plus =
LR(f(paste0(BB,"+N7+",v), d, ep), f(paste0(BB,"+N7+",v,"+",N8v), d, ep)),
                     HR = hr(mv, v)) }
  out }
R$sym_os <- sym(); R$sym_rfs <- sym(ep = "rfs")
show_sym <- function(S, lab) { pr("-- %s --", lab)
```

```
  pr("positivity over ref: chi2 %.2f dAIC %.1f | N8 over ref: chi2 %.2f (2 df) dAIC %.1f | subdivision over
positivity: chi2 %.2f p %.3f", S$positivity["chi"], S$positivity["dAIC"], S$N8["chi"], S$N8["dAIC"], S$sub-
div_over_pos["chi"], S$subdiv_over_pos["p"])
  for (v in c("lnr10","lodds","lognpos","npos")) { x <- S[[v]]
    pr("%-8s HR %.2f (%.2f-%.2f) | over ref chi2 %.2f dAIC %.1f | over positivity chi2 %.2f p %.4f | over N8
chi2 %.2f p %.4f | N8 over it chi2 %.2f (2df) p %.3f | positivity over it p %.3f | subdivision over positiv-
ity+it chi2 %.2f p %.3f",
       v, x$HR["hr"], x$HR["lo"], x$HR["hi"], x$over_ref["chi"], x$over_ref["dAIC"], x$over_pos["chi"],
x$over_pos["p"], x$over_N8["chi"], x$over_N8["p"], x$N8_over["chi"], x$N8_over["p"], x$pos_over["p"], x$sub-
div_over_pos_plus["chi"], x$subdiv_over_pos_plus["p"]) } }
show_sym(R$sym_os, "OS"); show_sym(R$sym_rfs, "RFS")


## Functional form
ff <- list()
for (ep in c("os","rfs")) { lin <- f(paste0(B,"+lnr10"), a, ep); sp <- f(paste0(B,"+",SP), a, ep); kL4 <-
quantile(np$lnr, c(.05,.35,.65,.95)); sp4 <- f(paste0(B,"+rcs(lnr,c(",paste(kL4,collapse=","),"))"), a, ep)
  m7 <- f(paste0(B,"+N7"), a, ep); m8 <- f(paste0(B,"+N8"), a, ep)
  linC <- f(paste0(B,"+npos"), a, ep); spC <- f(paste0(B,"+",SPC), a, ep); lg <- f(paste0(B,"+lognpos"), a,
ep)
  ff[[ep]] <- list(lnr_nonlin3 = LR(lin, sp), lnr_nonlin4 = LR(lin, sp4), AIC_lin = AIC(lin), AIC_sp =
AIC(sp),
    spline_over_N8 = LR(m8, f(paste0(B,"+N8+",SP), a, ep)), N8_over_spline = LR(sp, f(paste0(B,"+",SP,"+N8"),
a, ep)), pos_over_spline = LR(sp, f(paste0(B,"+",SP,"+N7"), a, ep)),
    spline_over_pos = LR(m7, f(paste0(B,"+N7+",SP), a, ep)), subdiv_over_pos_spline =
LR(f(paste0(B,"+N7+",SP), a, ep), f(paste0(B,"+N7+",SP,"+N8"), a, ep)),
    count_nonlin = LR(linC, spC), dAIC_count_lin = AIC(linC)-AIC(f(B,a,ep)), dAIC_count_sp = AIC(spC)-
AIC(f(B,a,ep)), dAIC_count_log = AIC(lg)-AIC(f(B,a,ep)),
    lnr_over_logcount = LR(lg, f(paste0(B,"+lognpos+lnr10"), a, ep)), logcount_over_lnr =
LR(f(paste0(B,"+lnr10"), a, ep), f(paste0(B,"+lnr10+lognpos"), a, ep)),
    lnr_over_splinecount = LR(spC, f(paste0(B,"+",SPC,"+lnr10"), a, ep)), splinecount_over_N8 = LR(m8,
f(paste0(B,"+N8+",SPC), a, ep)), knots_lnr = kL, knots_count = kC)
  pr("%s: LNR nonlinearity p %.3f (3 knots) / %.3f (4 knots); spline LNR over N8 chi2 %.2f (%d df) p %.4f; N8
over spline p %.3f; positivity over spline p %.3f; subdivision over positivity+spline p %.3f | count nonline-
arity p %.3f; dAIC count linear %.1f spline %.1f log %.1f; LNR over log-count chi2 %.2f p %.4f; log-count
over LNR p %.3f",
     ep, ff[[ep]]$lnr_nonlin3["p"], ff[[ep]]$lnr_nonlin4["p"], ff[[ep]]$spline_over_N8["chi"],
ff[[ep]]$spline_over_N8["df"], ff[[ep]]$spline_over_N8["p"], ff[[ep]]$N8_over_spline["p"],
ff[[ep]]$pos_over_spline["p"], ff[[ep]]$subdiv_over_pos_spline["p"],
     ff[[ep]]$count_nonlin["p"], ff[[ep]]$dAIC_count_lin, ff[[ep]]$dAIC_count_sp, ff[[ep]]$dAIC_count_log,
ff[[ep]]$lnr_over_logcount["chi"], ff[[ep]]$lnr_over_logcount["p"], ff[[ep]]$logcount_over_lnr["p"]) }
R$form <- ff
sp3 <- f(paste0(B,"+",SP)); xs <- c(0,0.05,0.1,0.15,0.2,0.3,0.4,0.5); Xp <- Hmisc::rcspline.eval(xs, knots =
kL, inclx = TRUE)
bb <- coef(sp3)[grep("rcs", names(coef(sp3)))]; V <- vcov(sp3)[grep("rcs", names(coef(sp3))), grep("rcs",
names(coef(sp3)))]
lp <- as.numeric(Xp %*% bb); lp <- lp - lp[1]; D <- sweep(Xp, 2, Xp[1,]); se <- sqrt(rowSums((D %*% V) * D))
R$form$spline_curve <- data.frame(lnr = xs, HR = exp(lp), lo = exp(lp-1.96*se), hi = exp(lp+1.96*se), linear
= exp(coef(f(paste0(B,"+lnr10")))["lnr10"]*10*xs)); print(round(R$form$spline_curve, 2))


## Table 2: reference model + LNR; univariable (origin-stratified)
mL <- f(paste0(B,"+lnr10")); s <- summary(mL); R$table2 <- list(multi = cbind(s$conf.int[, c(1,3,4)], p =
s$coef[,5]))
uni <- t(sapply(c("age","sexf","Tf","gradef","R1","pni","lvi","ca","N7","lnr10"), function(v) { m <-
coxph(as.formula(paste0("Surv(os_time,os_event)~",v,"+strata(origin)")), a); s <- summary(m);
c(s$conf.int[1,c(1,3,4)], p = s$coef[1,5]) }))
R$table2$uni <- uni; print(round(R$table2$multi, 3)); print(round(uni, 3))
R$table2$events_per_param <- c(ref = sum(a$os_event)/length(coef(f(B))), lnr =
sum(a$os_event)/length(coef(mL)), N8 = sum(a$os_event)/length(coef(f(paste0(B,"+N8")))), both =
sum(a$os_event)/length(coef(f(paste0(B,"+N8+lnr10")))))


## N2 versus N1 within node-positive patients; LNR within node-positive; precision
R$n2n1 <- list(unadj = hr(coxph(Surv(os_time,os_event)~N8, np), "N8N2"), strat =
hr(coxph(Surv(os_time,os_event)~N8+strata(origin), np), "N8N2"), full = hr(f(paste0(B,"+N8"), np), "N8N2"),
  lnr_within = hr(f(paste0(B,"+lnr10"), np), "lnr10"), lnr_over_n2n1 = LR(f(paste0(B,"+N8"), np),
f(paste0(B,"+N8+lnr10"), np)), n2n1_over_lnr = LR(f(paste0(B,"+lnr10"), np), f(paste0(B,"+lnr10+N8"), np)),
  n_pos = nrow(np), deaths_pos = sum(np$os_event), rfs_pos = sum(np$rfs_event), p_N2 = mean(np$N8=="N2"))
R$n2n1$mdh80 <- exp((qnorm(.975)+qnorm(.80))/sqrt(R$n2n1$deaths_pos * R$n2n1$p_N2 * (1-R$n2n1$p_N2)));
R$n2n1$mdh90 <- exp((qnorm(.975)+qnorm(.90))/sqrt(R$n2n1$deaths_pos * R$n2n1$p_N2 * (1-R$n2n1$p_N2)))
pr("N2 vs N1: unadjusted %.2f (%.2f-%.2f); stratified %.2f (%.2f-%.2f); full %.2f (%.2f-%.2f) | within N+:
LNR HR %.2f, LNR over N2vsN1 chi2 %.2f p %.4f, N2vsN1 over LNR p %.3f | deaths among N+ %d; minimal detecta-
ble HR (80%%) %.2f, (90%%) %.2f",
   R$n2n1$unadj[1],R$n2n1$unadj[2],R$n2n1$unadj[3], R$n2n1$strat[1],R$n2n1$strat[2],R$n2n1$strat[3],
R$n2n1$full[1],R$n2n1$full[2],R$n2n1$full[3], R$n2n1$lnr_within[1], R$n2n1$lnr_over_n2n1["chi"],
R$n2n1$lnr_over_n2n1["p"], R$n2n1$n2n1_over_lnr["p"], R$n2n1$deaths_pos, R$n2n1$mdh80, R$n2n1$mdh90)


## Kaplan-Meier descriptives (Figure S2)
km <- function(fo, d) summary(survfit(fo, data = d))$table
R$km <- list(N8 = km(Surv(os_time,os_event)~N8, a)[, c("records","events","median","0.95LCL","0.95UCL")],
logrank_N8 = 1-pchisq(survdiff(Surv(os_time,os_event)~N8, a)$chisq, 2), logrank_N1N2 = 1-pchisq(sur-
vdiff(Surv(os_time,os_event)~N8, np)$chisq, 1))
a$band3 <- cut(a$lnr, c(-Inf, 0, 0.2, Inf), labels = c("0", ">0-0.2", ">0.2")); R$km$band3 <-
km(Surv(os_time,os_event)~band3, a)[, c("records","events","median","0.95LCL","0.95UCL")]; R$km$logrank_band3
<- 1-pchisq(survdiff(Surv(os_time,os_event)~band3, a)$chisq, 2)
print(R$km$N8); print(R$km$band3); pr("log-rank N0/N1/N2 p %.2g; N1 vs N2 p %.3f; LNR bands p %.2g",
R$km$logrank_N8, R$km$logrank_N1N2, R$km$logrank_band3)


## Table S3: optimism-corrected C-index (Harrell), 1000 bootstrap resamples.
## For each resample b: fit each model on the resample, C_train[b] = C on the resample, C_test[b] = C of the
same
## fit on the original cohort; optimism = mean(C_train - C_test); corrected C = apparent C - optimism.
```

```
## Differences versus the N-category model: corrected difference = apparent difference - mean of the paired
## optimism difference; the 95% CI and P come from the paired per-resample differences in C_test.
cid <- function(m, d, rhs, tv = "os_time") { X <- model.matrix(as.formula(paste0("~",
gsub("\\+strata\\(origin\\)","",rhs))), d)[,-1,drop=FALSE]; lp <- as.numeric(X %*% coef(m)[colnames(X)])
  as.numeric(concordance(Surv(d[[tv]], d$os_event) ~ lp, reverse = TRUE)$concordance) }
cboot <- function(d, BB, Dv, tv = "os_time", NB = 1000, seed = 20260905, ref = "+ N0/N1/N2 (AJCC 8th)") {
  g <- function(rhs, dd = d) coxph(as.formula(paste0("Surv(", tv, ",os_event)~", rhs)), dd)
  rhsv <- sapply(Dv, function(x) paste0(BB, if (nchar(x)) paste0("+", x) else ""))
  app <- sapply(rhsv, function(r) cid(g(r), d, r, tv))
  set.seed(seed); tr <- te <- matrix(NA_real_, NB, length(Dv), dimnames = list(NULL, names(Dv)))
  for (b in 1:NB) { db <- d[sample(nrow(d), replace = TRUE), ]
    for (j in seq_along(Dv)) { mb <- tryCatch(g(rhsv[j], db), error = function(e) NULL)
      if (!is.null(mb)) { tr[b, j] <- tryCatch(cid(mb, db, rhsv[j], tv), error = function(e) NA_real_); te[b,
j] <- tryCatch(cid(mb, d, rhsv[j], tv), error = function(e) NA_real_) } } }
  opt <- colMeans(tr - te, na.rm = TRUE); corr <- app - opt
  pv <- function(x) 2 * min(mean(x < 0, na.rm = TRUE), mean(x > 0, na.rm = TRUE))
  t(sapply(names(Dv), function(k) { dte <- te[, k] - te[, ref]; dopt <- (tr[, k] - tr[, ref]) - dte
    c(apparent = unname(app[k]), optimism = unname(opt[k]), C = unname(corr[k]), diff = unname((app[k] -
app[ref]) - mean(dopt, na.rm = TRUE)),
      dlo = unname(quantile(dte, .025, na.rm = TRUE)), dhi = unname(quantile(dte, .975, na.rm = TRUE)), p =
pv(dte)) })) }
D <- c("Clinicopathological model only"="", "+ Nodal positivity"="N7", "+ N0/N1/N2 (AJCC 8th)"="N8", "+
log(positive nodes + 1)"="lognpos", "+ Lymph node ratio"="lnr10", "+ Log odds of positive nodes"="lodds")
R$tableS3 <- cboot(a, B, D)
print(round(R$tableS3, 4))


cat("\n==================== 3.3 Influence of nodal yield ====================\n")
BE <- paste0(B, "+ln_total")
R$eln <- list(sym_os = sym(BB = BE), sym_rfs = sym(BB = BE, ep = "rfs"),
  eln_hr = list(alone = hr(f(BE), "ln_total"), with_N7 = hr(f(paste0(BE,"+N7")), "ln_total"), with_N8 =
hr(f(paste0(BE,"+N8")), "ln_total"), with_lnr = hr(f(paste0(BE,"+lnr10")), "ln_total"), with_lodds =
hr(f(paste0(BE,"+lodds")), "ln_total")),
  N8_hr_with_eln = summary(f(paste0(BE,"+N8")))$conf.int[c("N8N1","N8N2"), c(1,3,4)], lnr_hr_with_eln =
hr(f(paste0(BE,"+lnr10")), "lnr10"),
  spline = list(spline_over_N8 = LR(f(paste0(BE,"+N8")), f(paste0(BE,"+N8+",SP))), N8_over_spline =
LR(f(paste0(BE,"+",SP)), f(paste0(BE,"+",SP,"+N8"))), pos_over_spline = LR(f(paste0(BE,"+",SP)),
f(paste0(BE,"+",SP,"+N7")))),
  log_eln = list(lnr_over_N8 = LR(f(paste0(B,"+log(ln_total)+N8")), f(paste0(B,"+log(ln_total)+N8+lnr10"))),
N8_over_lnr = LR(f(paste0(B,"+log(ln_total)+lnr10")), f(paste0(B,"+log(ln_total)+lnr10+N8")))),
  eln12 = list(lnr_over_N8 = LR(f(paste0(B,"+I(ln_total>=12)+N8")), f(paste0(B,"+I(ln_to-
tal>=12)+N8+lnr10"))), N8_over_lnr = LR(f(paste0(B,"+I(ln_total>=12)+lnr10")), f(paste0(B,"+I(ln_to-
tal>=12)+lnr10+N8")))),
  lnr_x_eln = LR(f(paste0(B,"+N8+lnr10+ln_total")), f(paste0(B,"+N8+lnr10*ln_total"))), spearman = c(lnr =
cor(a$lnr, a$ln_total, method="spearman"), lodds = cor(a$lodds, a$ln_total, method="spearman"), npos =
cor(a$ln_pos, a$ln_total, method="spearman")))
show_sym(R$eln$sym_os, "ELN-adjusted, OS"); show_sym(R$eln$sym_rfs, "ELN-adjusted, RFS")
pr("ELN HR per node: alone %.3f (%.3f-%.3f) p %.3f; with N8 %.3f (%.3f-%.3f); with LNR %.3f (%.3f-%.3f) |
spline: LNR over N8 p %.3f, N8 over spline p %.3f, positivity over spline p %.3f | log ELN: %.3f/%.3f |
ELN>=12: %.3f/%.3f | LNR x ELN p %.2f",
   R$eln$eln_hr$alone[1],R$eln$eln_hr$alone[2],R$eln$eln_hr$alone[3],R$eln$eln_hr$alone[4],
R$eln$eln_hr$with_N8[1],R$eln$eln_hr$with_N8[2],R$eln$eln_hr$with_N8[3],
R$eln$eln_hr$with_lnr[1],R$eln$eln_hr$with_lnr[2],R$eln$eln_hr$with_lnr[3],
   R$eln$spline$spline_over_N8["p"], R$eln$spline$N8_over_spline["p"], R$eln$spline$pos_over_spline["p"],
R$eln$log_eln$lnr_over_N8["p"], R$eln$log_eln$N8_over_lnr["p"], R$eln$eln12$lnr_over_N8["p"],
R$eln$eln12$N8_over_lnr["p"], R$eln$lnr_x_eln["p"])


## Period
per <- list()
for (k in c("2013-2016","2017-2024","2018-2024")) { d <- droplevels(subset(a, if (k=="2013-2016") yr<2017
else if (k=="2017-2024") yr>=2017 else yr>=2018)); dn <- droplevels(subset(d, N8!="N0"))
  BB <- if (k=="2013-2016") "age+pni+strata(origin)" else B    # reduced model in the small early subset
  per[[k]] <- list(n = nrow(d), deaths = sum(d$os_event), rfs = sum(d$rfs_event), npos = nrow(dn), deaths_pos
= sum(dn$os_event), N8 = table(d$N8), eln_median = median(d$ln_total), eln_q = quantile(d$ln_total,
c(.25,.75)),
    sym = sym(d, BB), sym_rfs = if (k!="2013-2016") sym(d, BB, "rfs") else NULL,
    spline = if (k!="2013-2016") list(N8_over_spline = LR(f(paste0(BB,"+",SP), d), f(paste0(BB,"+",SP,"+N8"),
d)), pos_over_spline = LR(f(paste0(BB,"+",SP), d), f(paste0(BB,"+",SP,"+N7"), d)), spline_over_N8 =
LR(f(paste0(BB,"+N8"), d), f(paste0(BB,"+N8+",SP), d))) else NULL,
    rho_lnr_count_pos = cor(dn$lnr, dn$ln_pos, method = "spearman"), lnr_within = lapply(split(dn, dn$N8),
function(g) c(n = nrow(g), median = median(g$lnr), q1 = quantile(g$lnr,.25), q3 = quantile(g$lnr,.75), min =
min(g$lnr), max = max(g$lnr), gt02 = mean(g$lnr > 0.2), eln_median = median(g$ln_total))),
    n2n1 = if (nrow(dn) > 20) hr(f(paste0(BB,"+N8"), dn), "N8N2") else NA, lnr_within_hr = if (nrow(dn) > 20)
hr(f(paste0(BB,"+lnr10"), dn), "lnr10") else NA,
    lnr_over_n2n1 = if (nrow(dn) > 20) LR(f(paste0(BB,"+N8"), dn), f(paste0(BB,"+N8+lnr10"), dn)) else NA,
n2n1_over_lnr = if (nrow(dn) > 20) LR(f(paste0(BB,"+lnr10"), dn), f(paste0(BB,"+lnr10+N8"), dn)) else NA)
  S <- per[[k]]$sym; pr("%s: n %d deaths %d N+ %d (deaths %d) ELN %d | positivity chi2 %.2f | subdivision
over pos chi2 %.2f p %.3f | LNR over pos chi2 %.2f p %.3f | LNR over N8 chi2 %.2f p %.3f | N8 over LNR p %.3f
| positivity over LNR p %.3f | subdivision over pos+LNR p %.3f | rho(LNR,count|N+) %.2f",
     k, nrow(d), sum(d$os_event), nrow(dn), sum(dn$os_event), median(d$ln_total), S$positivity["chi"], S$sub-
div_over_pos["chi"], S$subdiv_over_pos["p"], S$lnr10$over_pos["chi"], S$lnr10$over_pos["p"],
S$lnr10$over_N8["chi"], S$lnr10$over_N8["p"], S$lnr10$N8_over["p"], S$lnr10$pos_over["p"], S$lnr10$sub-
div_over_pos_plus["p"], per[[k]]$rho_lnr_count_pos)
  if (!is.null(per[[k]]$spline)) pr("   spline: N8 over spline p %.3f; positivity over spline p %.3f; spline
over N8 p %.3f", per[[k]]$spline$N8_over_spline["p"], per[[k]]$spline$pos_over_spline["p"],
per[[k]]$spline$spline_over_N8["p"])
  print(per[[k]]$lnr_within) }
R$period <- per
R$period$interaction <- list(lnr_x_period = LR(f(paste0(B,"+lnr10+I(yr>=2017)")),
f(paste0(B,"+lnr10*I(yr>=2017)"))), lnr_x_period_inN8 = LR(f(paste0(B,"+N8+lnr10+I(yr>=2017)")),
f(paste0(B,"+N8+lnr10*I(yr>=2017)"))))
R$period$drop_early_pos <- { d <- droplevels(subset(a, !(yr<2017 & N8!="N0"))); list(n = nrow(d), lnr_over_N8
= LR(f(paste0(B,"+N8"), d), f(paste0(B,"+N8+lnr10"), d)), N8_over_lnr = LR(f(paste0(B,"+lnr10"), d),
f(paste0(B,"+lnr10+N8"), d))) }
```

```
pr("LNR x period p %.2f (alone) / %.2f (in N8 model); dropping the %d early node-positive patients: LNR over
N8 chi2 %.2f p %.3f", R$period$interaction$lnr_x_period["p"], R$period$interaction$lnr_x_period_inN8["p"],
sum(a$yr<2017 & a$N8!="N0"), R$period$drop_early_pos$lnr_over_N8["chi"], R$pe-
riod$drop_early_pos$lnr_over_N8["p"])
R$period$drop_early_pos_eln15 <- { d <- droplevels(subset(a, ln_total >= 15 & !(yr<2017 & N8!="N0"))); list(n
= nrow(d), n_dropped = sum(a$ln_total >= 15 & a$yr<2017 & a$N8!="N0"), lnr_over_N8 = LR(f(paste0(B,"+N8"),
d), f(paste0(B,"+N8+lnr10"), d))) }
pr(">=15 examined nodes without the %d early node-positive patients (n=%d): LNR over N8 chi2 %.2f p %.3f",
R$period$drop_early_pos_eln15$n_dropped, R$period$drop_early_pos_eln15$n, R$pe-
riod$drop_early_pos_eln15$lnr_over_N8["chi"], R$period$drop_early_pos_eln15$lnr_over_N8["p"])


## Baseline hazard stratified by period of resection (second revision); examined-node count with period;
Spearman CIs
BP <- paste0(B, "+strata(period)"); BPC <- paste0(B, "+period")
R$period$stratified <- list(os = sym(a, BP), rfs = sym(a, BP, "rfs"), os_covariate = sym(a, BPC), rfs_covari-
ate = sym(a, BPC, "rfs"), period_hr_in_lnr_model = hr(f(paste0(BPC,"+lnr10")), "period2017-2024"),
  eln_hr = sapply(c(alone = "", with_N8 = "+N8", with_lnr = "+lnr10"), function(ex) rbind(no_period =
hr(f(paste0(B,"+ln_total",ex)), "ln_total"), period_covariate = hr(f(paste0(BPC,"+ln_total",ex)), "ln_to-
tal"), period_stratum = hr(f(paste0(BP,"+ln_total",ex)), "ln_total")), simplify = FALSE),
  eln_adjusted_os = sym(a, paste0(BP,"+ln_total")), eln_adjusted_rfs = sym(a, paste0(BP,"+ln_total"), "rfs"),
  pni_x_period = LR(f(paste0(BP,"+lnr10")), f(paste0(BP,"+lnr10+pni:period"))), pni_hr_by_period = sap-
ply(c("2013-2016","2017-2024"), function(k) hr(f(paste0(B,"+lnr10"), subset(a, period == k)), "pni")))
for (ep in c("os","rfs")) { S <- R$period$stratified[[ep]]
  pr("period-stratified %s: positivity chi2 %.2f | subdivision over pos chi2 %.2f p %.3f | LNR over pos chi2
%.2f p %.4f | LNR over N8 chi2 %.2f p %.4f | N8 over LNR chi2 %.2f p %.3f | positivity over LNR chi2 %.2f p
%.4f | subdivision over pos+LNR p %.3f | LNR HR %.2f (%.2f-%.2f)",
     ep, S$positivity["chi"], S$subdiv_over_pos["chi"], S$subdiv_over_pos["p"], S$lnr10$over_pos["chi"],
S$lnr10$over_pos["p"], S$lnr10$over_N8["chi"], S$lnr10$over_N8["p"], S$lnr10$N8_over["chi"],
S$lnr10$N8_over["p"], S$lnr10$pos_over["chi"], S$lnr10$pos_over["p"], S$lnr10$subdiv_over_pos_plus["p"],
S$lnr10$HR[1], S$lnr10$HR[2], S$lnr10$HR[3]) }
pr("period as covariate, OS: subdivision over pos p %.3f | LNR over N8 chi2 %.2f p %.4f | N8 over LNR p %.3f
| period HR %.2f (%.2f-%.2f)", R$period$stratified$os_covariate$subdiv_over_pos["p"], R$period$strati-
fied$os_covariate$lnr10$over_N8["chi"], R$period$stratified$os_covariate$lnr10$over_N8["p"], R$period$strati-
fied$os_covariate$lnr10$N8_over["p"], R$period$stratified$period_hr_in_lnr_model[1], R$period$stratified$pe-
riod_hr_in_lnr_model[2], R$period$stratified$period_hr_in_lnr_model[3])
for (k in names(R$period$stratified$eln_hr)) { e <- R$period$stratified$eln_hr[[k]]; pr("ELN HR per node %-
8s: no period %.3f (%.3f-%.3f) | period covariate %.3f (%.3f-%.3f) | period stratum %.3f (%.3f-%.3f)", k,
e[1,1], e[1,2], e[1,3], e[2,1], e[2,2], e[2,3], e[3,1], e[3,2], e[3,3]) }
S <- R$period$stratified$eln_adjusted_os; pr("ELN-adjusted + period stratum, OS: subdivision over pos chi2
%.2f p %.3f | LNR over pos chi2 %.2f p %.3f | LNR over N8 chi2 %.2f p %.3f | N8 over LNR p %.3f | PNI x pe-
riod p %.3f", S$subdiv_over_pos["chi"], S$subdiv_over_pos["p"], S$lnr10$over_pos["chi"],
S$lnr10$over_pos["p"], S$lnr10$over_N8["chi"], S$lnr10$over_N8["p"], S$lnr10$N8_over["p"], R$period$strati-
fied$pni_x_period["p"])
set.seed(20260905)
R$period$spearman_ci <- lapply(c("2013-2016","2017-2024"), function(k) { d <- subset(a, period == k & N8 !=
"N0"); r <- cor(d$lnr, d$ln_pos, method = "spearman")
  bs <- replicate(5000, { i <- sample(nrow(d), replace = TRUE); if (length(unique(d$lnr[i])) < 2 ||
length(unique(d$ln_pos[i])) < 2) NA else cor(d$lnr[i], d$ln_pos[i], method = "spearman") })
  fz <- tanh(atanh(r) + c(-1,1)*1.96*1.06/sqrt(nrow(d)-3)); c(n = nrow(d), rho = r, boot_lo = unname(quan-
tile(bs,.025,na.rm=TRUE)), boot_hi = unname(quantile(bs,.975,na.rm=TRUE)), fisher_lo = fz[1], fisher_hi =
fz[2]) }); names(R$period$spearman_ci) <- c("2013-2016","2017-2024")
for (k in names(R$period$spearman_ci)) { z <- R$period$spearman_ci[[k]]; pr("Spearman rho(LNR, count | N+)
%s: n %d rho %.2f, bootstrap 95%% CI %.2f to %.2f (Fisher z %.2f to %.2f)", k, z["n"], z["rho"],
z["boot_lo"], z["boot_hi"], z["fisher_lo"], z["fisher_hi"]) }


## Reclassification at LNR 0.18 (Figure 2, Table S4)
np$hi <- np$lnr > 0.18
R$reclass <- list(table = table(N8 = np$N8, LNR_gt_018 = np$hi))
grpstat <- function(s) c(n = nrow(s), pos_median = median(s$ln_pos), eln_median = median(s$ln_total), eln_q1
= quantile(s$ln_total,.25), eln_q3 = quantile(s$ln_total,.75), eln_min = min(s$ln_total), eln_max =
max(s$ln_total), lnr_median = median(s$lnr), deaths = sum(s$os_event), median_os = unname(summary(sur-
vfit(Surv(os_time,os_event)~1, s))$table["median"]))
R$reclass$N1_to_high <- grpstat(subset(np, N8=="N1" & hi)); R$reclass$N2_to_low <- grpstat(subset(np,
N8=="N2" & !hi)); R$reclass$N1_conc <- grpstat(subset(np, N8=="N1" & !hi)); R$reclass$N2_conc <- grpstat(sub-
set(np, N8=="N2" & hi))
disc <- subset(np, (N8=="N1" & hi) | (N8=="N2" & !hi)); R$reclass$logrank <- 1-pchisq(sur-
vdiff(Surv(os_time,os_event)~N8, disc)$chisq, 1); R$reclass$n_disc <- nrow(disc); R$reclass$deaths_disc <-
sum(disc$os_event)
R$reclass$tableS4 <- disc[order(disc$N8, disc$ln_total), c("N8","origin","ln_to-
tal","ln_pos","lnr","yr","os_event","os_time")]
print(R$reclass$table); print(rbind(N1_to_high = R$reclass$N1_to_high, N2_to_low = R$reclass$N2_to_low,
N1_conc = R$reclass$N1_conc, N2_conc = R$reclass$N2_conc)); pr("discordant: n %d deaths %d log-rank p %.3f",
nrow(disc), sum(disc$os_event), R$reclass$logrank); print(R$reclass$tableS4, row.names = FALSE)


cat("\n==================== 3.4 Exploratory and sensitivity analyses ====================\n")
## Table S5: threshold scan (exploratory)
R$tableS5 <- t(sapply(seq(0.05, 0.45, by = 0.05), function(cut) { a$X <- as.numeric(a$lnr > cut); s <-
hr(f(paste0(B,"+X"), a), "X"); L1 <- LR(f(paste0(B,"+N8"), a), f(paste0(B,"+N8+X"), a)); L2 <-
LR(f(paste0(B,"+N8"), a, "rfs"), f(paste0(B,"+N8+X"), a, "rfs"))
  c(cut = cut, n_above = sum(a$X), HR = s[1], lo = s[2], hi = s[3], p = s[4], chi_over_N8 = L1["chi"],
p_over_N8 = L1["p"], p_over_N8_rfs = L2["p"]) })); print(round(R$tableS5, 3))
## Table S6: tumor origin
d3 <- droplevels(subset(a, origin != "Duodenum"))
R$tableS6 <- list(by_origin = t(sapply(levels(d3$origin), function(o) { d <- droplevels(subset(a, origin ==
o)); c(n = nrow(d), deaths = sum(d$os_event), npos = sum(d$N7=="N+"), hr(coxph(Surv(os_time,os_event)~lnr10,
d), "lnr10")) })),
  interaction = sapply(c("lnr10","lodds","N8"), function(v) LR(f(paste0(COV,"+origin+",v), d3),
f(paste0(COV,"+origin*",v), d3))["p"]),
  ratio_of_HR = { mi <- f(paste0(COV,"+origin*lnr10"), d3); s <- summary(mi)$conf.int; s[grep(":lnr10",
rownames(s)), c(1,3,4)] })
print(R$tableS6)
## Table S1: sensitivity analyses — primary test (LNR over N8), reverse test, subdivision over positivity
```

```
after_rec <- a$recur==1 & !is.na(a$adj_start) & !is.na(a$recur_date) & as.Date(a$adj_start) >= as.Date(a$re-
cur_date); after_rec[is.na(after_rec)] <- FALSE
a$adj_strict <- as.integer(a$adj==1 & !after_rec & !(!is.na(a$adj_days) & a$adj_days > 180))
a$ca37 <- factor(ifelse(is.na(a$ca199),"not measured",ifelse(a$ca199>37,">37","<=37")), lev-
els=c("<=37",">37","not measured")); a$ca100 <- factor(ifelse(is.na(a$ca199),"not meas-
ured",ifelse(a$ca199>100,">100","<=100")), levels=c("<=100",">100","not measured"))
a$logca <- ifelse(is.na(a$ca199), 0, log(a$ca199)); a$ca_nm <- as.integer(is.na(a$ca199)); a$bil2 <- as.inte-
ger(a$tbil > 2)
a$Tf8 <- a$Tf; ph <- a$origin == "Pancreatic head"; a$Tf8[ph] <- factor(ifelse(a$size_x[ph] > 4 | a$pT[ph] ==
4, "pT3-4", "pT1-2"), levels = levels(a$Tf))   # size-based 8th-edition T for the pancreatic head
SS <- list(
  list("Primary analysis", a, B),
  list("Excluding duodenal adenocarcinoma (n=143)", subset(a, origin != "Duodenum"), B),
  list("N1/N2 cut-point 1-3 / >=4 applied to all origins", a, B, "N8u"),
  list("Excluding death within 90 days (n=146)", subset(a, death90 == 0), B),
  list("Examined nodes >=12 (n=113)", subset(a, ln_total >= 12), B),
  list("Examined nodes >=15 (n=96)", subset(a, ln_total >= 15), B),
  list("Resected 2017 onward (n=113)", subset(a, yr >= 2017), B),
  list("Resected 2018 onward (n=102)", subset(a, yr >= 2018), B),
  list("Upfront surgery only (n=142)", subset(a, neoadj != 1 | is.na(neoadj)), B),
  list("Excluding adenosquamous carcinoma (n=143)", subset(a, histo_txt != "adenosquamous carcinoma"), B),
  list("Pancreatobiliary origins only (n=112)", subset(a, origin %in% c("Pancreatic head","Distal bile
duct")), B),
  list("Tumor origin as a covariate instead of stratum", a, paste0(COV,"+origin")),
  list("Reduced covariate set (pT, PNI)", a, "Tf+pni+strata(origin)"),
  list("Lymphovascular invasion added", a, paste0(B,"+lvi")),
  list("Calendar year of resection added", a, paste0(B,"+yr")),
  list("Examined-node count added", a, BE),
  list("Baseline hazard stratified by period of resection", a, BP),
  list("Examined-node count added, stratified by period", a, paste0(BP,"+ln_total")),
  list("pT omitted", a, sub("Tf\\+","",B)),
  list("Origin-specific pT terms", a, paste0(sub("Tf\\+","",COV),"+Tf:origin+strata(origin)")),
  list("Pancreatic head pT re-derived from tumor size (8th edition)", a, sub("Tf","Tf8",B)),
  list("CA19-9 at 37 U/mL", a, sub("\\+ca\\+","+ca37+",B)),
  list("CA19-9 at 100 U/mL", a, sub("\\+ca\\+","+ca100+",B)),
  list("log CA19-9, continuous", a, sub("\\+ca\\+","+logca+ca_nm+",B)),
  list("CA19-9 omitted", a, sub("\\+ca\\+","+",B)),
  list("Preoperative bilirubin >2 mg/dL added", a, paste0(B,"+bil2")),
  list("Postoperative treatment added (strict definition)", a, paste0(B,"+adj_strict")),
  list("Postoperative treatment added (as recorded; submitted model)", a, paste0(B,"+adj")),
  list("Survivors censored at last clinic visit (submitted rule)", a, B, "N8", "os_time_visit"))
S4 <- list()
for (x in SS) { d <- droplevels(x[[2]]); BB <- x[[3]]; N8v <- if (length(x) >= 4) x[[4]] else "N8"; tv <- if
(length(x) >= 5) x[[5]] else "os_time"
  if (nlevels(d$origin) < 2) BB <- gsub("\\+strata\\(origin\\)", "", BB)
  g <- function(rhs) coxph(as.formula(paste0("Surv(", tv, ",os_event)~", rhs)), d)
  m7 <- g(paste0(BB,"+N7")); m8 <- g(paste0(BB,"+",N8v)); mL <- g(paste0(BB,"+lnr10")); h <- hr(mL, "lnr10")
  S4[[x[[1]]]] <- c(n = nrow(d), deaths = sum(d$os_event), HR = h[1], lo = h[2], hi = h[3], subdiv_chi =
LR(m7, m8)["chi"], subdiv_p = LR(m7, m8)["p"], lnr_over_N_chi = LR(m8,
g(paste0(BB,"+",N8v,"+lnr10")))["chi"], lnr_over_N_p = LR(m8, g(paste0(BB,"+",N8v,"+lnr10")))["p"],
    N_over_lnr_p = LR(mL, g(paste0(BB,"+lnr10+",N8v)))["p"], subdiv_over_pos_lnr_p =
LR(g(paste0(BB,"+N7+lnr10")), g(paste0(BB,"+N7+lnr10+",N8v)))["p"]) }
## time-dependent postoperative treatment (OS only; start date)
tdd <- subset(a, adj == 0 | !is.na(adj_days)); tdd$adj_t <- ifelse(tdd$adj == 1, pmax(tdd$adj_days,
1)/30.4375, NA); tdd$adj_t[!is.na(tdd$adj_t) & tdd$adj_t >= tdd$os_time] <- NA
sp <- tmerge(tdd[, c("id","os_time","os_event")], tdd[, c("id","os_time","os_event")], id = id, dth =
event(os_time, os_event)); sp <- tmerge(sp, tdd[, c("id","adj_t")], id = id, adjtd = tdc(adj_t))
sp <- merge(sp, tdd[, setdiff(names(tdd), c("os_time","os_event"))], by = "id")
g <- function(rhs) coxph(as.formula(paste0("Surv(tstart,tstop,dth)~", rhs)), data = sp); m7 <-
g(paste0(B,"+adjtd+N7")); m8 <- g(paste0(B,"+adjtd+N8")); mL <- g(paste0(B,"+adjtd+lnr10")); h <- hr(mL,
"lnr10")
S4[["Postoperative treatment as a time-dependent covariate"]] <- c(n = nrow(tdd), deaths = sum(tdd$os_event),
HR = h[1], lo = h[2], hi = h[3], subdiv_chi = LR(m7, m8)["chi"], subdiv_p = LR(m7, m8)["p"], lnr_over_N_chi =
LR(m8, g(paste0(B,"+adjtd+N8+lnr10")))["chi"], lnr_over_N_p = LR(m8, g(paste0(B,"+adjtd+N8+lnr10")))["p"],
N_over_lnr_p = LR(mL, g(paste0(B,"+adjtd+lnr10+N8")))["p"], subdiv_over_pos_lnr_p =
LR(g(paste0(B,"+adjtd+N7+lnr10")), g(paste0(B,"+adjtd+N7+lnr10+N8")))["p"])
R$adj_hr <- list(strict = hr(f(paste0(B,"+adj_strict+lnr10")), "adj_strict"), recorded =
hr(f(paste0(B,"+adj+lnr10")), "adj"), timedep = hr(mL, "adjtd"), n_strict = sum(a$adj_strict), n_recorded =
sum(a$adj), n_after_rec = sum(after_rec & a$adj==1), n_late = sum(a$adj==1 & !is.na(a$adj_days) & a$adj_days
> 180),
  uptake_by_N8 = tapply(a$adj, a$N8, mean), uptake_by_origin_N7 = tapply(a$adj, list(a$origin, a$N7), mean),
start_days = quantile(a$adj_days[a$adj==1], c(.5,.25,.75), na.rm = TRUE), ph_adj_model =
cox.zph(f(paste0(B,"+adj+lnr10")))$table["adj","p"])
## multiple imputation for CA19-9 (m = 20)
a$na_H <- nelsonaalen(a, os_time, os_event)
imp_dat <- a[, c("ca199","age","sexf","Tf","gradef","R1","pni","origin","lnr10","N8","N7","ln_to-
tal","tbil","os_event","na_H","rfs_event")]; imp_dat$logca <- log(imp_dat$ca199); imp_dat$ca199 <- NULL;
imp_dat$tbil <- log(imp_dat$tbil)
meth <- make.method(imp_dat); meth["logca"] <- "pmm"; imp <- mice(imp_dat, m = 20, method = meth, seed =
20260905, printFlag = FALSE)
il <- complete(imp, "long", include = TRUE); il$ca_i <- factor(ifelse(exp(il$logca) > 200, ">200", "<=200"),
levels = c("<=200", ">200")); il$os_time <- rep(a$os_time, 21); il$os_event <- rep(a$os_event, 21); il$n2 <-
as.integer(il$N8 == "N2"); imp2 <- as.mids(il)
Bi <- "age+sexf+Tf+gradef+R1+pni+ca_i+strata(origin)"; W <- function(rhs) with(imp2, coxph(as.for-
mula(paste0("Surv(os_time,os_event)~", rhs))))
pl <- summary(pool(W(paste0(Bi,"+lnr10")))); r <- pl[pl$term=="lnr10",]
R$mi <- list(lnr_hr = c(exp(r$estimate), exp(r$estimate-1.96*r$std.error), exp(r$estimate+1.96*r$std.error)),
lnr_over_N8_p = D1(W(paste0(Bi,"+N8+lnr10")), W(paste0(Bi,"+N8")))$result[1,4], N8_over_lnr_p =
D1(W(paste0(Bi,"+lnr10+N8")), W(paste0(Bi,"+lnr10")))$result[1,4], subdiv_p = D1(W(paste0(Bi,"+N7+n2")),
W(paste0(Bi,"+N7")))$result[1,4])
S4[["CA19-9 multiply imputed (m = 20)"]] <- c(n = nrow(a), deaths = sum(a$os_event), HR = R$mi$lnr_hr[1], lo
= R$mi$lnr_hr[2], hi = R$mi$lnr_hr[3], subdiv_chi = NA, subdiv_p = R$mi$subdiv_p, lnr_over_N_chi = NA,
lnr_over_N_p = R$mi$lnr_over_N8_p, N_over_lnr_p = R$mi$N8_over_lnr_p, subdiv_over_pos_lnr_p = NA)
R$tableS1 <- do.call(rbind, S4); print(round(R$tableS1, 3))
```

```
## CA19-9 descriptives (Methods / response)
R$ca <- list(days_before_op = quantile(a$ca_days_before_op, c(.5,.25,.75,0,1), na.rm=TRUE), drainage =
mean(a$drain_yn==1), bil_gt2 = mean(a$tbil>2), bil_days = median(a$tbil_days_before_op, na.rm=TRUE),
rho_ca_bil = cor(a$ca199, a$tbil, method="spearman", use="complete.obs"),
  gt37 = mean(a$ca199>37, na.rm=TRUE), gt100 = mean(a$ca199>100, na.rm=TRUE), gt200 = mean(a$ca199>200,
na.rm=TRUE), ca_hr_in_jaundiced = hr(f(paste0(B,"+lnr10"), subset(a, tbil>2)), "ca>200"), ca_hr_in_nonjaun-
diced = hr(f(paste0(B,"+lnr10"), subset(a, tbil<=2)), "ca>200"), n_jaundiced = sum(a$tbil>2))
## pT by edition; proportional hazards; LODDS-ELN
R$pt <- list(edition_by_origin = table(a$origin, a$ed), interaction_p = LR(f(paste0(COV,"+origin"), d3),
f(paste0(sub("Tf\\+","",COV),"+origin*Tf"), d3))["p"])
print(R$pt$edition_by_origin); pr("pT x origin interaction p %.3f", R$pt$interaction_p)
R$ph <- list(lnr_model = cox.zph(f(paste0(B,"+lnr10")))$table, N8_model_global =
cox.zph(f(paste0(B,"+N8")))$table["GLOBAL","p"]); print(R$ph$lnr_model)
## Multiplicity note
R$multiplicity <- list(primary_p = R$sym_os$lnr10$over_N8["p"], bonferroni_10 = min(1,
10*R$sym_os$lnr10$over_N8["p"]), bonferroni_20 = min(1, 20*R$sym_os$lnr10$over_N8["p"]))


cat("\n==================== Transparency: submitted vs revised specifications (response letter)
====================\n")
spec <- function(lab, d, BB, tv, N8v) { g <- function(rhs, dd = d) coxph(as.formula(paste0("Surv(", tv,
",os_event)~", rhs)), dd); m0 <- g(BB); m7 <- g(paste0(BB,"+N7")); m8 <- g(paste0(BB,"+",N8v)); mL <-
g(paste0(BB,"+lnr10")); h <- hr(mL, "lnr10")
  c(n = nrow(d), deaths = sum(d$os_event), pos_chi = LR(m0, m7)["chi"], subdiv_chi = LR(m7, m8)["chi"], sub-
div_p = LR(m7, m8)["p"], lnr_hr = h[1], lnr_lo = h[2], lnr_hi = h[3], lnr_over_N_chi = LR(m8,
g(paste0(BB,"+",N8v,"+lnr10")))["chi"], lnr_over_N_p = LR(m8, g(paste0(BB,"+",N8v,"+lnr10")))["p"],
N_over_lnr_p = LR(mL, g(paste0(BB,"+lnr10+",N8v)))["p"],
    n2n1 = hr(g(paste0(BB,"+",N8v), droplevels(d[d[[N8v]] != "N0",])), paste0(N8v,"N2"))[1]) }
Bsub <- paste0(B, "+adj"); a146 <- subset(a, death90 == 0)
R$transparency <- rbind(`Submitted (postoperative treatment in model; last clinic visit; 90-day death ex-
cluded; uniform N1/N2 cut-point)` = spec("sub", a146, Bsub, "os_time_visit", "N8u"),
  `Postoperative treatment removed` = spec("b", a146, B, "os_time_visit", "N8u"), `+ censoring at last date
known alive` = spec("c", a146, B, "os_time", "N8u"),
  `+ 90-day death included` = spec("d", a, B, "os_time", "N8u"), `+ organ-specific N definition (final)` =
spec("e", a, B, "os_time", "N8"))
print(round(R$transparency, 3))
## C-index difference (LNR vs N category) under the originally submitted specification, same optimism correc-
tion
R$cindex_submitted <- cboot(a146, Bsub, c("+ N0/N1/N2 (AJCC 8th)"="N8u", "+ Lymph node ratio"="lnr10"), tv =
"os_time_visit", ref = "+ N0/N1/N2 (AJCC 8th)")
print(round(R$cindex_submitted, 4))
## C-index of the reference, N-category and LNR models under each specification, by the present procedure and
by the originally submitted procedure
## (submitted procedure: each model fitted on a resample and evaluated on the original cohort; C = mean over
resamples; no optimism subtraction)
cv1 <- function(d, BB, Dv, tv, NB = 1000, seed = 20260905, ref = "N8") {
  g <- function(rhs, dd) coxph(as.formula(paste0("Surv(", tv, ",os_event)~", rhs)), dd)
  cidv <- function(m, dd) { lp <- predict(m, newdata = dd, type = "lp"); as.numeric(concord-
ance(Surv(dd[[tv]], dd$os_event) ~ lp, reverse = TRUE)$concordance) }
  set.seed(seed); bc <- matrix(NA_real_, NB, length(Dv), dimnames = list(NULL, names(Dv)))
  for (b in 1:NB) { db <- d[sample(nrow(d), replace = TRUE), ]
    for (j in seq_along(Dv)) { mb <- tryCatch(g(paste0(BB, if (nchar(Dv[j])) paste0("+", Dv[j]) else ""),
db), error = function(e) NULL)
      if (!is.null(mb)) bc[b, j] <- tryCatch(cidv(mb, d), error = function(e) NA_real_) } }
  dif <- bc[, "lnr10"] - bc[, ref]
  c(ref = mean(bc[,"ref"], na.rm=TRUE), N = mean(bc[,ref], na.rm=TRUE), lnr = mean(bc[,"lnr10"], na.rm=TRUE),
diff = mean(dif, na.rm=TRUE), dlo = unname(quantile(dif,.025,na.rm=TRUE)), dhi = unname(quan-
tile(dif,.975,na.rm=TRUE)), p = 2*min(mean(dif<0,na.rm=TRUE), mean(dif>0,na.rm=TRUE))) }
specs <- list(`Submitted specification` = list(a146, Bsub, "os_time_visit", "N8u"), `Postoperative treatment
removed` = list(a146, B, "os_time_visit", "N8u"), `+ censoring at last date known alive` = list(a146, B,
"os_time", "N8u"),
  `+ 90-day death included` = list(a, B, "os_time", "N8u"), `+ organ-specific N definition (final)` = list(a,
B, "os_time", "N8"))
R$cindex_by_spec <- t(sapply(specs, function(s) { Dv <- c(ref = "", N8 = s[[4]], lnr10 = "lnr10"); r <-
cboot(s[[1]], s[[2]], Dv, tv = s[[3]], ref = "N8"); v <- cv1(s[[1]], s[[2]], Dv, tv = s[[3]])
  c(n = nrow(s[[1]]), present_ref = r["ref","C"], present_N = r["N8","C"], present_lnr = r["lnr10","C"], pre-
sent_diff = r["lnr10","diff"], present_dlo = r["lnr10","dlo"], present_dhi = r["lnr10","dhi"], present_p =
r["lnr10","p"], apparent_ref = r["ref","apparent"], optimism_ref = r["ref","optimism"],
    submitted_ref = v["ref"], submitted_N = v["N"], submitted_lnr = v["lnr"], submitted_diff = v["diff"],
submitted_dlo = v["dlo"], submitted_dhi = v["dhi"], submitted_p = v["p"]) }))
print(round(R$cindex_by_spec, 3))


write_json(R, "output/results_v3.json", auto_unbox = TRUE, digits = 8, pretty = TRUE, force = TRUE)
saveRDS(R, "output/results_v3.rds")
cat("\n== Session information ==\n"); print(sessionInfo())
```
